# Forward flight of birds revisited. Part 1: aerodynamics and performance

**DOI:** 10.1098/rsos.140248

**Published:** 2014-10-15

**Authors:** G. Iosilevskii

**Affiliations:** Faculty of Aerospace Engineering, Technion, Haifa 32000, Israel

**Keywords:** aerodynamics, flight performance, flapping flight, propulsion efficiency

## Abstract

This paper is the first part of the two-part exposition, addressing performance and dynamic stability of birds. The aerodynamic model underlying the entire study is presented in this part. It exploits the simplicity of the lifting line approximation to furnish the forces and moments acting on a single wing in closed analytical forms. The accuracy of the model is corroborated by comparison with numerical simulations based on the vortex lattice method. Performance is studied both in tethered (as on a sting in a wind tunnel) and in free flights. Wing twist is identified as the main parameter affecting the flight performance—at high speeds, it improves efficiency, the rate of climb and the maximal level speed; at low speeds, it allows flying slower. It is demonstrated that, under most circumstances, the difference in performance between tethered and free flights is small.

## 2. Introduction

Aerodynamics and flight mechanics of flapping flight have drawn research attention since the beginning of the aviation era. The complexity of aerodynamic models involved progressively increased, until recent advances in computing power have made full Reynolds-averaged Navier-Stokes simulations within reach [1]. It seems, however, that in the race for fidelity, a few fundamental problems became buried under excessive details. Two of these problems, performance and short-term dynamic stability of birds in forward flight, are revisited in this study with the simplest aerodynamic model feasible. The performance is addressed in this part; the short term dynamic stability is addressed in part 2 [2]. The aerodynamic model, serving both parts, is constructed herein.

By ‘simplest model feasible’, we understand a model that can furnish the aerodynamic loads in a closed analytical form, and is accurate enough to capture their behaviour in flapping flight. Taking the cue from [3], we construct this model in the framework of the basic lifting line theory. The present model differs from that of [3] in allowing additional degrees of freedom for the wings' motion, in furnishing the forces acting on a single wing as well as their first moments, and in remarkable simplicity of its final expressions. Using the vortex lattice method to provide the reference, the model is shown in §§4.5 and 5.2 to be accurate enough for all the purposes of this two-part study. It intrinsically limits it, however, to those flyers that generate lift without flow separation—that is, sufficiently large birds and bats (but not insects).

A wing invariably twists during flapping. The effect of twist on flight performance was addressed in many papers (e.g. [1,3,4]), but the twist never received the credit of being the most important parameter affecting the performance. It could have happened because of the historical definition of the propulsion efficiency: the ratio of the average power made good (the product of force produced by the wings in the direction of flight and airspeed) and the average power spent (see for example [4,5]). This definition ignores the double role played by flapping wings in flight. Stopping the wings makes the force generated by them in the direction of flight negative—in fact, this force becomes drag, parasite and induced combined. In comparison, it is commonly accepted that stopping the propeller of an aeroplane makes its thrust vanish—the drag of the aeroplane's wings is considered an inseparable part of the aeroplane's total drag. As propulsion efficiency of a propeller is defined irrespective of the aerodynamic characteristics of the aeroplane it propels, so the propulsion efficiency of flapping wings should be defined irrespective of the aerodynamic characteristics of the bird in non-flapping flight. Propulsion efficiency and its dependence on twist are addressed in §5.1.

A common measure of performance of a fixed wing aeroplane is the specific excess power [6]—essentially, it is the maximal sustained rate of climb at a given airspeed. We did not see the use of this measure in relation to flight performance of birds. Specific excess power of an aeroplane is limited by the engine power throughout the flight envelope. Specific excess power of a bird is also limited by the maximal power the bird can generate, but only at high speeds. Increasing the power at low speeds stalls the wings, putting an additional limit on performance. Specific excess power of a bird and its dependence on twist are addressed in §5.4.

Bird's body invariably pitches and heaves during flapping. Effects of the body motion on performance are addressed in §§6.1–6.3.

## 3. Kinematics

Consider a simplified symmetric bird in symmetric flight with constant velocity *v*. The mass of the bird is *m*; the density of the air in which it flies is *ρ*; acceleration of gravity is *g*; the length of a single wing (the semi-span) is *s*; its area is *S*; the aspect ratio of the two wings is *A*=2*s*^2^/*S*. *s*, *v*, *vs*, *s*/*v*, *v*/*s*, *ρsv*^2^, *ρs*^2^*v*^2^, *ρSv*^2^, *ρSv*^2^*s* and *ρSv*^3^ will serve as convenient units of length, velocity, circulation, time, frequency, force per unit span, moment per unit span, force, moment and power, respectively. Note that although *S* is half the quantity commonly used as the wing area, the units of force and power are standard. Use of dimensionless quantities is implicitly understood hereafter. Should a dimensional quantity (other than *ρ*, *g*, *m*, *s*, *S* and *v*) be required, it will be marked by an asterisk. A list of nomenclature can be found in table 1.

**Table 1.** Nomenclature.

fundamental quantities	
*g*	acceleration of gravity
*m*	mass
*S*	area of one wing (right or left)
*s*	semi-span
*v*	flight velocity
*ρ*	air density
fundamental units	
*s*	length
*v*	velocity
*υs*	circulation
*s*/*v*	time
*v*/*s*	frequency
*ρsv*^2^	force per unit span
*ρs*^2^*v*^2^	moment per unit span
*ρSv*^2^	force
*ρSv*^2^*s*	moment
*ρSv*^3^	power
non-dimensional quantities	
*A*	aspect ratio
*A*_1_,*A*_2_,…	functions of the aspect ratio defined in (3.1)
*a*	lift slope coefficient of the wing's section (2*π*)
*a*_1_,*a*_3_,…	Fourier coefficients in the expansion of *Γ*
*c*	local chord
*D*,*D*_0_,*D*_i_	drag—total, parasite and induced
*d*	parameter defined in (5.15)
*E*_*s*_	specific cost of locomotion
**e**_*x*_, **e**_*y*_, **e**_*z*_	basis vectors of the frame C, following the bird along a straight path
$e_{x}^{L},e_{y}^{L},e_{z}^{L}$	basis vectors of the frame L, rigidly attached to the local chord
*F*_*ex*_	specific excess thrust
**f**	force per unit span
*H*_1_	function of the aspect ratio and of the twist parameter defined in (5.23)
*h*	vertical displacement of the wing's root from a straight path
*I*_11_,*I*_13_,…	standard integrals defined in (A 4); *I*_11_=−1/3 and *I*_13_=−1/5
*K*_1_,…,*K*_5_	functions of the aspect ratio defined in (3.28) and (3.29)
*k*_1_,…,*k*_5_	reduced forms of *K*_1_,…,*K*_5_ defined in (3.31)
*k*_*ϕ*_, $k_{\dot{\phi }}$	constants characterizing the flapping pattern
*k*_*ω*_	coefficient in (5.29)
*L*, $L_{\max}$	lift, maximal lift
*M*_*x*_	twice the flapping moment of the right wing
*M*_*y*_	twice the pitching moment of the right wing
**m**	moment per unit span
*P*_*s*_	specific excess power
*t*	time
*T*,*T*′,*T*_*ex*_	thrust, proper thrust and excess thrust
**v**^*C*^	velocity of a point on the wing relative to C
$\dot{W},{\dot{W}}_{\mathrm{mg}},{\dot{W}}_{\mathrm{mg}}^{\prime }$	power, power made good, and proper power made good
*α*, *α*_0_, *α*_1_	angle of attack, its value at the root, its increase along the span
*α*_*c*_	maximal angle of attack along the span
*α*_*g*_, *α*_*g*0_, *α*_*g*1_	twist angle, its value at the root, its increase along the span
*α*_i_	induced angle of attack
*Γ*	circulation
*δ*_*nm*_	Kronecker's delta
*ε*	twist parameter defined in (5.10)
*η*	propulsion efficiency
*θ*	spanwise variable
λ	sweep angle
*τ*	body angle relative to the average flight path
*ϕ*, *ϕ*_0_	flapping angle and flapping amplitude
*ω*	angular frequency
special symbols	
…^′^	adjusted for drag in the adjoint flight
…*	non-fundamental dimensional quantity
…^*C*^	reference frame C
…^*L*^	reference frame L
$\overset{\cdot }{.\;.\;.}$	derivative with respect to time
$\begin{array}{c} \text{---} \end{array}$	adjoint flight
〈…〉	average over a single period

**Figure 1.**
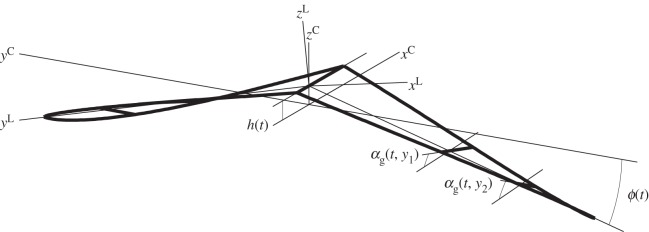
Reference frames and wing motion parameters. The wing is allowed to heave (*h*), flap (*ϕ*), sweep (λ), pitch (*τ*) and twist (*α*_*g*_) about the quarter-chord line. The twist shown is highly exaggerated; sweep and pitch are not shown. Frame L is rigidly connected to a local section of the wing; the axes shown correspond to the mid span of the right wing. Frame C is an inertial reference frame that follows the bird along a straight path at the distance *h* beneath the wing.

Each wing is allowed to flap, sweep fore and aft, twist, heave and pitch. It is assumed that the wing twists in such a way that its sections do not deform and remain parallel to each other; moreover, the twist axis crosses all sections at their respective quarter-chord points and remains straight at all times. The sweep angle of the twist axis is λ (positive aft), flapping angle is *ϕ* (positive down); the twist angle is *α*_*g*_ (positive for leading edge up); pitch angle relative to the average flight path is *τ* (positive for nose up); vertical translation of the twist axis is *h* (figure 1). It is assumed that the twist varies linearly along the span, with

3.1 $$\alpha_{g}=\alpha_{g0}+\alpha_{g1}y,$$

where *y*∈(0,1) is the spanwise coordinate; the description here pertains to the right wing. The twist can be active (through muscle contraction) or passive (through aerodynamic twisting moment); no attempt is made to model its intricate details.

Two right-handed Cartesian reference frames will be used. Frame L is rigidly connected to a local chord; its *y*-axis coincides with the twist axis of the right wing; its *x*-axis points backwards parallel to the chord; its *z*-axis points upwards and its origin rests in the symmetry plane of the bird (where the two wings meet). Frame C follows the bird with constant velocity (*v*) along a straight path; its *x*-axis points backwards along that path; its *y*-axis points right and its *z*-axis points upwards, through the origin of L. Unit vectors along the axes of L are $e_{x}^{L}$, $e_{y}^{L}$, $e_{z}^{L}$; unit vectors along the axes of C are $e_{x}=e_{x}^{C}$, $e_{y}=e_{y}^{C}$, $e_{z}=e_{z}^{C}$. Heave is manifested in time dependence of the distance *h* between the origins of L and C. Rotation from C to L is a series of four Euler's rotations: about the *y*-axis through angle *τ*; about the new *z*-axis through angle −λ; about the new *x*-axis through angle −*ϕ* and about the new *y*-axis through angle *α*_*g*_. A comparable approach can be found in [5].

Explicit expressions for the components of $e_{x}^{L}$, $e_{y}^{L}$ and $e_{z}^{L}$ in C are lengthy, and hence are not written here for the general case. In the particular case where all angles are small when compared with unity

3.2 $$\begin{array}{rl} e_{x}^{L} & =e_{x}-\lambda e_{y}-(\alpha_{g}+\tau )e_{z}+\cdots , \end{array}$$

3.3 $$\begin{array}{rl} e_{y}^{L} & =\lambda e_{x}+e_{y}-\phi e_{z}+\cdots \end{array}$$

3.4 $$\begin{array}{rl} \text{and}\;e_{z}^{L} & =(\alpha_{g}+\tau )e_{x}+\phi e_{y}+e_{z}+\cdots , \end{array}$$

where the ellipses stand for the higher-order terms with respect to angles and their time derivatives. Concurrently, the velocity of a point on the wing relative to C is

3.5 $$v^{C}=\dot{\lambda }ye_{x}-\dot{\lambda }xe_{y}+(\dot{h}-\dot{\phi }y-({\dot{\alpha }}_{g}+\dot{\tau })x)e_{z}+\cdots ,$$

where *x* and *y* are (by interpretation) the distances from the twist and flapping axes respectively, and an over-dot stands for derivative with respect to (dimensionless) time.

## 4. Aerodynamics

### 4.1 Assumptions

It is assumed that *A*^−1^, *α*_*g*_, *τ*, $\dot{h}$, *ϕ*, $\dot{\phi }$, λ and $\dot{\lambda }$ are small when compared with unity. The first assumption underlies the lifting line theory; the remaining assumptions underlie linearization. For the sake of simplicity of the following discussion, it is assumed that *α*_*g*_, *τ*, $\dot{h}$ and $\dot{\phi }$ are of comparable magnitudes, say Δ. The magnitude of λ is assumed not to exceed *A*^−1^ and *ϕ*; $\dot{\lambda }$ is assumed to be a second-order quantity with respect to *ϕ*, Δ and their products.

It is postulated that a vortical wake exists past the wing, starting at the trailing edge and extending to infinity. It is assumed that the vorticity is constant along that portion of the wake adjacent to the trailing edge that affects the flow over the wing. This assumption implies that the flapping frequency *ω* is sufficiently small; it is plausible—in fact, it will be shown by example—that *ω* can be of the order of unity. The aerodynamic model developed herein is coherent only in the leading order with respect to *ϕ*, Δ, and their products with *ω*.

It is assumed that the wing has an elliptical plan-form with chord length prescribed by

4.1 $$c=c_{0}\sqrt{1-y^{2}}=\frac{8}{\pi A}\sqrt{1-y^{2}};$$

*c*_0_=8/*π*A, because *S*=*s*^2^*c*_0_*π*/4 and *A*=2*s*^2^/*S* by definition. Exploiting the symmetry of the problem, the range of *y* in all subsequent equations is extended to (−1, 1); negative values corresponding to the left wing.

### 4.2 Fundamentals

As already mentioned in §2, the aerodynamic model for this study is based on the classical (quasi-steady) lifting line theory ([7, p. 586]). In brief, this theory associates the lift of a wing section, represented by the right-hand side of the following equation, with the lift of an equivalent vortex, represented by the left-hand side

4.2 $$\Gamma =\frac{1}{2}ca(\alpha -\alpha_{i})\;\mathrm{for}\;\mathrm{each}\;y\in (-1,1).$$

**Figure 2.**
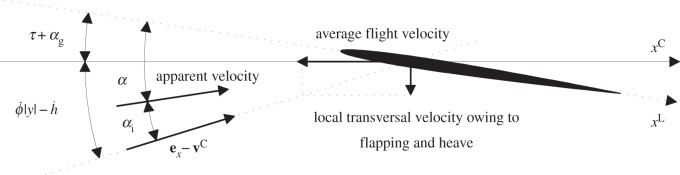
Constituents of the local angle of attack.

*Γ* is the circulation of that vortex, *a*=2*π* is the lift-slope coefficient of the wing section, *α* is the effective angle of attack the wing section relative to unperturbed fluid (that can be considered known), and *α*_i_ is the angle of attack induced by the wake (figure 2). The closure of (4.2) is obtained by relating *α*_i_ with *Γ* by Biot–Savart's law ([7], p. 94) which transforms (4.2) into an integro-differential equation for *Γ*. Once solved, the aerodynamic loads follow by quadratures.

The non-intuitive elements in this paradigm are the angles *α* and *α*_i_. In the lifting-line limit, the wing reduces to a vortex in the *y*-*z* plane whereas the wake reduces to the sheet of vortices starting at the wing vortex and extending to infinity in the positive *x*-direction [8]. Coherent with this model, *α* is approximated by its leading-order term (with respect to the aspect ratio), $\underset{x\to 0}{\lim}(e_{z}^{L}\cdot (e_{x}-v^{C})/|e_{x}-v^{C}|)$. For a non-cambered wing, it yields

4.3 $$\begin{array}{rl} & \alpha =\tau +\alpha_{g}+\dot{\phi }|y|-\dot{h}+\cdots =\alpha_{0}+\alpha_{1}|y|+\cdots , \end{array}$$

4.4 $$\begin{array}{rl} & \alpha_{0}=\alpha_{g0}+\tau -\dot{h} \end{array}$$

4.5 $$\begin{array}{rl} \mathrm{and}\; & \alpha_{1}=\alpha_{g1}+\dot{\phi }, \end{array}$$

by (3.1), (3.4) and (3.5); it can be extended ad hoc for a cambered wing by defining *α*_*g*_ as the angle between the *x*-axis of C and the zero-lift line (rather than the chord) of the respective section. *α*_0_ will be recognized as the spanwise uniform constituent of the angle of attack—it is associated with pitch and heave of the body and twist at the shoulder; *α*_1_ is the gradient of the angle of attack along the span—it is associated with the flapping rate and with spanwise-variable twist.

*α*_i_ is approximated by its leading-order term with respect to the aspect ratio as well—that is, the normal-to-the-wing (reduced) velocity component in the *y*-*z* plane induced by the wake [8]. Because *ϕ* is small when compared with unity, and the wake vorticity is supposedly constant along that portion of the wake that affects the flow about the wing (see §4.1),

4.6 $$\alpha_{i}=\frac{1}{4\pi }--\int_{-1}^{1}\frac{\partial \Gamma^{\prime }}{\partial y^{\prime }}\frac{dy^{\prime }}{y-y^{\prime }}+\cdots .$$

A combination of (4.2) and (4.6) leads to the well-known integrodifferential equation for *Γ*,

4.7 $$\Gamma +\frac{ac}{8\pi }--\int_{-1}^{1}\frac{\partial \Gamma^{\prime }}{\partial y^{\prime }}\frac{dy^{\prime }}{y-y^{\prime }}=\frac{1}{2}ac\alpha \;\mathrm{for}\;\mathrm{each}\;y\in (-1,1),$$

in which *α* is given by (4.3), *c* is given by (4.1), and, in general, *a*=2*π*. Its relevant solution is

4.8 $$\Gamma =4\sum_{n=1,3,\ldots }^{\infty }\;a_{n}\sin n\theta ,$$

where

4.9 $$\begin{array}{rl} \theta & =\cos^{-1}(-y), \end{array}$$

4.9 $$\begin{array}{rl} a_{2n-1} & =A_{2n-1}\left(\alpha_{0}\delta_{n1}-\frac{4}{\pi }\alpha_{1}I_{1,2n-1}\right), \end{array}$$

*δ*_*nm*_ is Kronecker's delta, $I_{mn}=\int_{\pi /2}^{\pi }\sin n\theta \;\sin m\theta \;\cos\theta d\theta$ are standard integrals, and

4.11 $$A_{n}=\frac{a}{\pi A+na}=\frac{2}{A+2n}.$$

Details can be found in appendix A. Explicit expression for *α*_i_,

4.12 $$\alpha_{i}=\frac{1}{4\pi vs}--\int_{0}^{\pi }\frac{\partial \Gamma^{\prime }}{\partial \theta^{\prime }}\frac{d\theta^{\prime }}{\cos\theta^{\prime }-\cos\theta }=\sum_{n=1,3,\ldots }^{\infty }\;\;na_{n}\frac{\sin n\theta }{\sin\theta },$$

follows (4.6) by (4.8) and (A 2).

### 4.3 Forces and moments

The force per unit span acting on a section of the right wing, **f**, and its couple about the origin of L, **m**, are

4.13 $$f=\Gamma {\left(e_{x}-v^{C}-\alpha_{i}e_{z}\right)}_{x=0}\times e_{y}^{L}$$

and

4.14 $$m=ye_{y}^{L}\times f.$$

With (3.3) and (3.5), they take on the explicit forms

4.15 $$f=\Gamma ((\dot{h}-\dot{\phi }y+\alpha_{i})e_{x}+\phi e_{y}+e_{z}+\cdots )$$

and

4.16 $$m=y\Gamma (e_{x}-(\lambda +\phi (\dot{h}-\dot{\phi }y+\alpha_{i}))e_{y}-e_{z}(\dot{h}-\dot{\phi }y+\alpha_{i})+\cdots ),$$

where the ellipses stand for higher-order terms in *ϕ* and Δ; they are omitted hereafter. Consequently,

4.17 $$\begin{array}{rl} L & =Ae_{z}\cdot \int_{0}^{1}f\;dy=A\int_{0}^{1}\Gamma dy, \end{array}$$

4.18 $$\begin{array}{rl} T & =-Ae_{x}\cdot \int_{0}^{1}f\;dy=A\int_{0}^{1}\left(\dot{\phi }y-\dot{h}-\alpha_{i}\right)\Gamma dy \end{array}$$

4.19 $$\begin{array}{rl} \mathrm{and}\;M_{y} & =Ae_{y}\cdot \int_{0}^{1}m\;dy=-A\int_{0}^{1}\left(\lambda +\phi (\dot{h}-\dot{\phi }y+\alpha_{i})\right)\Gamma y\;dy, \end{array}$$

are the lift, thrust and pitching moment (about the origin of L) of the two wings combined;

4.20 $$M_{x}=Ae_{x}\cdot \int_{0}^{1}m\;dy=A\int_{0}^{1}\Gamma y\;dy$$

is twice the rolling (flapping) moment acting on the right wing; and

4.21 $$\dot{W}=-A\int_{0}^{1}\underset{x\to 0}{\lim}v^{C}\cdot f\;dy=-A\int_{0}^{1}\left(\dot{h}-\dot{\phi }y\right)\Gamma dy=-\dot{h}L+\dot{\phi }M_{x}$$

is the power needed to move both wings. Because in the dimensionless representation, the power made good, ${\dot{W}}_{\mathrm{mg}}$, and the thrust, *T*, are equivalent, the conjunction of (4.21) and (4.18),

4.22 $${\dot{W}}_{\mathrm{mg}}=T=\dot{W}-D_{i},$$

where

4.23 $$D_{i}=A\int_{0}^{1}\alpha_{i}\Gamma dy$$

is coherent with the interpretation of *D*_i_ as the induced drag. In (4.19), the first term in the right-hand side, the one involving λ, reflects the contribution of the lift to the pitching moment; the remaining terms reflect the contribution of the thrust.

With (4.8)–(4.10), (4.12), (A 4) and (A 6), equations (4.17), (4.23), (4.19) and (4.20) take on the explicit forms

4.24 $$\begin{array}{rl} L & =\pi Aa_{1}=\pi AA_{1}\left(\alpha_{0}-\frac{4}{\pi }I_{11}\alpha_{1}\right), \end{array}$$

4.25 $$\begin{array}{rl} D_{i} & =\pi A\sum_{n=1,3,\ldots }^{\infty }\;\;na_{n}^{2}=\pi AA_{1}^{2}\alpha_{0}^{2}-8AA_{1}^{2}I_{11}\alpha_{0}\alpha_{1}+A\frac{16}{\pi }K_{2}\alpha_{1}^{2}, \end{array}$$

4.26 $$\begin{array}{rl} M_{x} & =-4A\sum_{n=1,3,\ldots }^{\infty }\;\;a_{n}I_{1n}=-4AA_{1}I_{11}\left(\alpha_{0}-\frac{4}{\pi }\frac{K_{1}}{A_{1}I_{11}}\alpha_{1}\right), \end{array}$$

4.27 $$\begin{array}{rl} M_{y} & =4A(\dot{h}\phi +\lambda )\sum_{n=1,3,\ldots }^{\infty }\;\;a_{n}I_{1n}+\frac{\pi }{4}A\phi \dot{\phi }(a_{1}+a_{3})+4A\phi \sum_{n=1,3,\ldots }^{\infty }\;\;\sum_{m=1,3,\ldots }^{\infty }\;\;ma_{n}a_{m}I_{mn}\\ & =4A(\dot{h}\phi +\lambda )\left(\alpha_{0}A_{1}I_{11}-\frac{4}{\pi }\alpha_{1}K_{1}\right)+\frac{\pi }{4}A\phi \dot{\phi }\left(\alpha_{0}A_{1}-\frac{4}{\pi }\alpha_{1}(A_{1}I_{11}+A_{3}I_{13})\right)\\ & \;+4\phi \alpha_{0}^{2}AA_{1}^{2}I_{11}-\frac{8}{\pi }\alpha_{0}\alpha_{1}\phi AA_{1}K_{4}+\frac{32}{\pi^{2}}\alpha_{1}^{2}A\phi K_{5}, \end{array}$$

where *A*_1_, *A*_3_, … have been defined in (4.11), whereas *I*_11_, *I*_13_, … can be found in (A 4)–(A 7). The four infinite sums in (4.25)–(4.27),

4.28 $$\begin{array}{rlrl} 2K_{1} & =\sum_{n=1,3,\ldots }^{\infty }\;\;A_{n}I_{1n}^{2}, & \;K_{2} & =\sum_{n=1,3,\ldots }^{\infty }\;\;nA_{n}^{2}I_{1n}^{2}, \end{array}$$

4.29 $$\begin{array}{rlrl} 2K_{4} & =2\sum_{n=1,3,\ldots }^{\infty }\;\;(1+n)A_{n}I_{1n}^{2}, & \;K_{5} & =2\sum_{n=1,3,\ldots }^{\infty }\;\;\sum_{m=1,3,\ldots }^{\infty }\;\;mA_{n}A_{m}I_{1m}I_{1n}I_{mn}, \end{array}$$

define four functions of the aspect ratio, *A*. Accurate low-order Padé approximations of these functions can be found in equations (B 1) and (B 3) of appendix B. Less accurate, but nonetheless useful, approximations can be obtained by setting

4.30 $$k_{1}\approx 1.29,\;k_{2}\approx 1.61,\;k_{4}\approx 2.29,\;k_{5}\approx 6.12$$

(independent of the aspect ratio) in

4.31 $$K_{1}=k_{1}A_{1}I_{11}^{2},\;K_{2}=k_{2}A_{1}^{2}I_{11}^{2},\;K_{4}=\frac{\pi^{2}}{32}k_{4}A_{1}\;\mathrm{and}\;K_{5}=-\frac{\pi^{2}}{32}k_{5}A_{1}^{2}I_{11}^{2}.$$

Despite its simple form (it equals −1/3 by (A 7)), *I*_11_ was left unevaluated in many of the subsequent equations to facilitate tracking of the particular terms.

Concluding this list is the explicit expression for the power required to move the wings,

4.32 $$\dot{W}=-4AA_{1}I_{11}\alpha_{0}\dot{\phi }+\frac{16}{\pi }AK_{1}\alpha_{1}\dot{\phi }-\pi AA_{1}\alpha_{0}\dot{h}+4AA_{1}I_{11}\alpha_{1}\dot{h};$$

it follows from (4.21) by (4.26) and (4.24). Explicit expression for the power made good, ${\dot{W}}_{\mathrm{mg}}=T$, immediately follows from (4.32) and (4.25) by (4.22), and hence is missed out.

### 4.4 Local angle of attack

The local angle of attack during flapping, *α*−*α*_i_, can be found directly, from (4.3) and (4.10)–(4.12):

4.33 $$\alpha -\alpha_{i}=\alpha_{0}(1-A_{1})+\alpha_{1}\left(\left|\cos\theta \right|+\frac{4}{\pi }\sum_{n=1,3,5,\ldots }^{\infty }\;\;nA_{n}I_{1n}\frac{\sin n\theta }{\sin\theta }\right);$$

or indirectly, from (4.2), (4.1), (4.8) and (4.10):

4.34 $$\alpha -\alpha_{i}=\frac{2\Gamma }{ca}=\frac{A}{2}\sum_{n=1,3,5,\ldots }^{\infty }\;\;a_{n}\frac{\sin n\theta }{\sin\theta }=\frac{A}{A+2}\alpha_{0}-\frac{2A}{\pi }\alpha_{1}\sum_{n=1,3,5,\ldots }^{\infty }\;\;A_{n}I_{1n}\frac{\sin n\theta }{\sin\theta }.$$

The equivalence between the two approaches is proved in appendix C.

Maximal angle of attack along the span, *α*_*c*_, will be needed in §5.3. It occurs at the wing tips, *θ*=0 and *θ*=*π*– this conjecture is proved in appendix D; consequently,

4.35 $$\alpha_{c}=\underset{\theta \to 0}{\lim}(\alpha -\alpha_{i})=\frac{AA_{1}}{2}\alpha_{0}+\alpha_{1}K_{3},$$

where the first term in the right-hand side follows the respective term in (4.34) by (4.11), whereas

4.36 $$K_{3}=-\frac{2A}{\pi }\underset{\theta \to 0}{\lim}\sum_{n=1,3,5,\ldots }^{\infty }\;\;A_{n}I_{1n}\frac{\sin n\theta }{\sin\theta }=-\frac{2A}{\pi }\sum_{n=1,3,5,\ldots }^{\infty }\;\;nA_{n}I_{1n}$$

is yet another function of the aspect ratio. Its Padé approximation can be found in (B 2).

### 4.5 Corroboration

**Figure 3.**
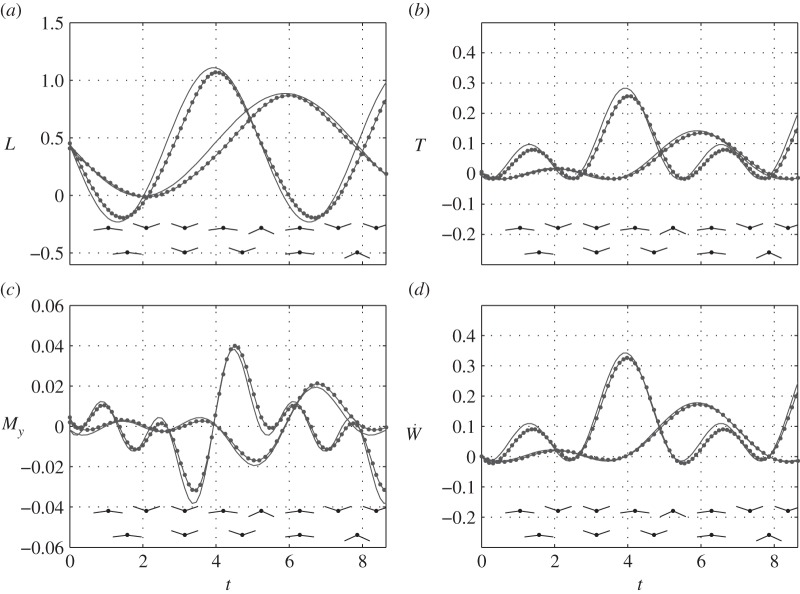
Lift, thrust, power and pitching moment of a harmonically flapping elliptic wing at *ω*=0.8 and *ω*=1.2. Dots represent numerical simulations; solid lines represent (4.24), (4.27), (4.32) and (4.22). Wing positions during flapping are shown, schematically, in two lines at the bottom of each figure: low frequency at the bottom line, high frequency at the top. Details can be found in appendix F.

**Figure 4.**
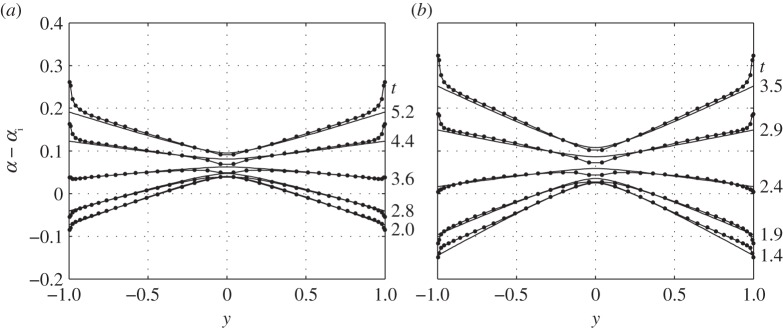
Effective angle of attack at the quarter-chord line of a flapping elliptic wing at (*a*) *ω*=0.8 and (*b*) *ω*=1.2 captured at several times during the cycle; these times appear to the right of each line. Dots represent numerical simulations; solid lines represent (4.34). Details can be found in appendix F.

The accuracy of (4.22), (4.24), (4.27), (4.32) and (4.34) was assessed by comparison with numerical solutions based on the vortex lattice method (appendix E). The cases chosen for this comparison represent a typical middle-sized bird (see appendix D of part 2): wing aspect ratio (*A*) between 6 and 16; flapping frequency (*ω*) between 0.8 and 1.6; flapping amplitude (*ϕ*_0_) of 15° and 30°;^1^ and twist distribution, characterized by *α*_*g*,0_=5° and *α*_*g*1_=−*εϕ*, where *ε* varies between 0.3 and 0.7.^2^ These parameters do not necessarily comply with all the assumptions of §4.1. Nevertheless, the accuracy of the present model seems to be fair and is certainly adequate for the purposes of this study (figures 3 and 4); more figures can be found in the electronic supplementary material. Recalling that the present model ignores unsteady and non-planar effects, its adequacy in predicting aerodynamic loads implies that at least in some cases, relevant to the flight of birds, these effects are indeed secondary to the effects of finite span. We will return to this point in §5.2.

## 5. Performance

### 5.1 Propulsion efficiency

The preceding sections were based on the traditional definitions of thrust and power made good. As already mentioned in §2, these definitions have to be modified for flapping flight, where the wings serve the dual role of providing lift and generating thrust. When the wings stop flapping, traditionally defined thrust becomes negative, it turns into induced drag—(see (4.22)). If it were a fixed wing aircraft, stopping the engine would have made its thrust vanish—the induced drag would have been an inseparable part of the aircraft drag, rather than part of its thrust. This inconsistency is removed here by differentiating between the proper thrust

5.1 $$T^{\prime }=T+{\bar{D}}_{i},$$

and the proper excess thrust,

5.2 $$T_{\mathrm{ex}}=T-D_{0}=T^{\prime }-({\bar{D}}_{i}+D_{0}).$$

*D*_0_ and ${\bar{D}}_{i}$ are the parasite drag and the induced drag in the matching (*adjoint*) non-flapping flight with the same average lift and the same velocity. The proper thrust *T*′ promptly vanishes when the wings stop flapping; the proper excess thrust *T*_ex_ vanishes when the proper thrust balances the total drag of the bird in the adjoint flight

5.3 $$\bar{D}=D_{0}+{\bar{D}}_{i}.$$

The propulsion efficiency can now be defined as the ratio of the average proper power made good, $\left\langle {\dot{W}}_{\mathrm{mg}}^{\prime }\rangle =\langle T^{\prime }\right\rangle$, to the average power needed to move the wings, $\left\langle \dot{W}\right\rangle$:

5.4 $$\eta =\frac{\langle T\rangle +{\bar{D}}_{i}}{\left\langle \dot{W}\right\rangle }=1-\frac{\langle D_{i}\rangle -{\bar{D}}_{i}}{\left\langle \dot{W}\right\rangle };$$

the angular brackets mark the respective period averages. The two forms follow by (5.1) and by the variant of (4.22), respectively. The first one is better suited for numerical computations, where the average thrust and the induced drag in the adjoint flight are readily available. The second one is clearer conceptually, as it explicitly associates the efficiency with the drag added by flapping, $\langle D_{i}\rangle -{\bar{D}}_{i}$. The ratio, $\left\langle T_{\mathrm{ex}}\rangle /\langle \dot{W}\right\rangle$, commonly used instead of (5.4)—for example, in [4,5]—is a viable figure of merit, but it is not efficiency.

Marking the respective parameters in the adjoint flight by over-bars, the adjoint flight is formally defined as the flight in which

5.5 $${\bar{\alpha }}_{0}=\langle \alpha_{0}\rangle ,\;{\bar{\alpha }}_{1}=\langle \alpha_{1}\rangle .$$

Accordingly,

5.6 $$\begin{array}{rl} \bar{L} & =\underset{\begin{array}{c} \alpha_{0}\to \langle \alpha_{0}\rangle \\ \alpha_{1}\to \langle \alpha_{1}\rangle \end{array}}{\lim}L=\pi AA_{1}\langle \alpha_{0}\rangle -4AA_{1}I_{11}\langle \alpha_{1}\rangle =\langle L\rangle \end{array}$$

5.7 $$\begin{array}{rl} \text{and}\;{\bar{D}}_{i} & =\underset{\begin{array}{c} \alpha_{0}\to \langle \alpha_{0}\rangle \\ \alpha_{1}\to \langle \alpha_{1}\rangle \end{array}}{\lim}D_{i}=\pi AA_{1}^{2}{\left\langle \alpha_{0}\right\rangle }^{2}-8AA_{1}^{2}I_{11}\langle \alpha_{0}\rangle \langle \alpha_{1}\rangle +A\frac{16}{\pi }K_{2}{\left\langle \alpha_{1}\right\rangle }^{2} \end{array}$$

by (4.24) and (4.25), where as

5.8 $$\eta =1-\frac{\pi AA_{1}^{2}\left(\langle \alpha_{0}^{2}\rangle -{\left\langle \alpha_{0}\right\rangle }^{2}\right)-8AA_{1}^{2}I_{11}(\langle \alpha_{0}\alpha_{1}\rangle -\langle \alpha_{0}\rangle \langle \alpha_{1}\rangle )+(16/\pi )AK_{2}\left(\langle \alpha_{1}^{2}\rangle -{\left\langle \alpha_{1}\right\rangle }^{2}\right)}{-4AI_{11}A_{1}\langle \alpha_{0}\dot{\phi }\rangle +(16/\pi )AK_{1}\langle \alpha_{1}\dot{\phi }\rangle -\pi AA_{1}\langle \alpha_{0}\dot{h}\rangle +4AA_{1}I_{11}\langle \alpha_{1}\dot{h}\rangle }$$

by (5.4), (4.32), (4.25), (4.22) and (5.7).

Three assumptions will greatly simplify the following discussion. The first one fixes the twist at the shoulder with

5.9 $${\dot{\alpha }}_{g0}=0.$$

The second one slaves the wing's twist to the flapping rate with

5.10 $$\alpha_{g1}=-\varepsilon \dot{\phi },$$

where *ε*∈(0,1) is a certain proportionality coefficient (as in [1,4]). As mentioned already in §3, it is immaterial whether this twist is achieved by active or passive means. In conjunction with (4.5), equation (5.10) implies

5.11 $$\alpha_{1}=(1-\varepsilon )\dot{\phi },$$

and, consequently,

5.12 $$\langle \alpha_{1}\rangle =\langle \alpha_{g1}\rangle =0;$$

by definition of the period average. The third assumption inhibits the rigid-body degrees of freedom of the bird with

5.13 $$\dot{h}=\dot{\tau }=0;$$

it is equivalent to placing the bird on a sting in a wind tunnel. This assumption will be released in §6.1. *A posteriori*, its effect on performance is small. Because of (5.9), (5.13) and (4.4),

5.14 $$\alpha_{0}=\langle \alpha_{0}\rangle =\alpha_{g0}+\tau .$$

Note that in the framework of the linear theory, the pitch (*τ*) and the spanwise-constant constituent of the twist (*α*_*g*,0_) are equivalent.

With (5.11)–(5.14), equations (5.8) and (4.32) reduce to

5.15 $$\eta =1-\frac{K_{2}\langle \alpha_{1}^{2}\rangle }{K_{1}\langle \alpha_{1}\dot{\phi }\rangle }=1-(1-\varepsilon )\frac{K_{2}}{K_{1}}=1-A_{1}(1-\varepsilon )\frac{k_{2}}{k_{1}}$$

and

**Figure 5.**
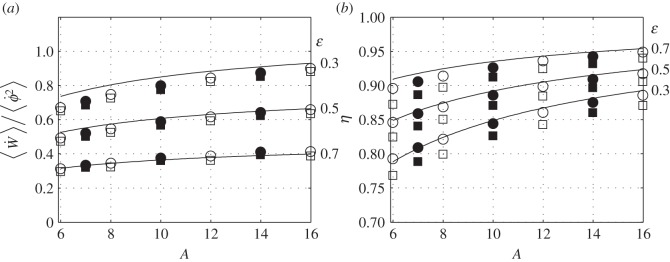
Average power (*a*) and efficiency (*b*) as functions of the aspect ratio for three values of the twist parameter. Solid lines represent equations (5.15) and (5.16); points represent numerical simulations with *ω*=0.8 (circles) and *ω*=1.2 (squares). Open symbols represent *ϕ*_0_=30°; closed symbols represent *ϕ*_0_=15°; details can be found in appendices E and F.

5.16 $$\langle \dot{W}\rangle =\frac{16}{\pi }AK_{1}\langle \alpha_{1}\dot{\phi }\rangle =\frac{16}{\pi }AK_{1}(1-\varepsilon )\langle {\dot{\phi }}^{2}\rangle =\frac{16k_{1}}{9\pi }AA_{1}(1-\varepsilon )\langle {\dot{\phi }}^{2}\rangle ;$$

expressions in the right-hand side follow those on their left by (A 7) and (4.31). Both parameters are shown in figure 5. The efficiency increases with increasing twist and aspect ratio; the power dramatically diminishes with increasing twist, and slightly increases with the aspect ratio.

Increasing the aspect ratio reduces the velocity induced by the wake; smaller induced velocity increases the undulatory constituent of the lift and reduces the respective constituent of the drag. Larger lift increases the power needed to flap the wings; smaller drag improves the efficiency. Increasing the twist reduces the undulatory constituents of the lift and the drag alike. Power required is associated with lift; power losses are associated with drag. Because lift is linear in the angle-of-attack and drag is quadratic, increasing the twist improves the efficiency.

### 5.2 Nonlinear effects

Computations of the average power and of the propulsion efficiency have been repeated using the vortex lattice method. Details of these computations can be found in appendices E and F; the results are shown in figure 5. The agreement between the numerical simulations and (5.16) is fair (figure 5*a*), regardless of the flapping amplitude and frequency. The agreement between the numerical simulations and (5.15) is fair at *ω*=0.8 (figure 5*b*), but worsens as the frequency increases.

A flapping wing leaves vortical wake behind it. Some of the vortices comprising the wake are associated with finiteness of the wing; others are associated with the wing's motion. Vortices of the first type are roughly parallel to the direction of the flow relative to the wing; they are created as long as the circulation varies along the span. Vortices of the second type are perpendicular to the direction of the flow; they are created any time there is a change in the flow about the wing. Both types of the vortices carry energy with them; energy loss to the wake is manifested as drag in the definition of the propulsion efficiency (5.4). Equation (5.15) accounts only for the energy carried away by vortices of the first type, and because the energy carried away by the vortices of the second type increases with the flapping frequency, the accuracy suffers.

The detrimental effect of increasing frequency on the propulsion efficiency offsets somewhat the beneficial effect of the increasing twist. For the same flapping amplitude and the same average power, increasing the twist parameter from 0.3 to 0.7, necessitates a 50% increase in the flapping frequency. This conjecture follows from (5.16), because in harmonic flapping $\langle {\dot{\phi }}^{2}\rangle =\phi_{0}^{2}\omega^{2}/2$. This increase in the twist parameter increases the propulsion efficiency from the bottom line in figure 5*b* to the top line—say, from 0.83 to 0.93 (at *A*=8). The 50% increase in the flapping frequency decreases the efficiency by the difference between the circles (*ω*=0.8) and the squares (*ω*=1.2) in figure 5*b*, approximately by 0.015. Hence, the decrease of the propulsion efficiency with frequency is a second-order effect—in fact, consistent with the assumptions underlying the present model.

The adjoint flight has been formally defined in (5.5) by specifying the angle of attack and the twist. In the framework of the linear theory, this definition yields $\bar{L}=\langle L\rangle$ by (4.24) (see (5.6)). However, in the framework of a nonlinear theory—as the one represented by our numerical solution—the lift in the adjoint flight also depends on the dihedral angle of the wings, $\bar{\phi }$. To obtain $\bar{L}=\langle L\rangle$, $|\bar{\phi }|$ should be, roughly, two-thirds of the flapping amplitude; stopping the wings at $\bar{\phi }=0$ yields $\bar{L}>\langle L\rangle$. We could have defined the adjoint flight differently, by specifying ${\bar{\alpha }}_{1}=\langle \alpha_{1}\rangle$, $\bar{L}=\langle L\rangle$ and $\bar{\phi }=0$. It would have changed nothing in the linear theory, but it would have implied ${\bar{\alpha }}_{0}<\langle \alpha_{0}\rangle$ (and hence $\bar{D}<\underset{\begin{array}{c} \alpha_{0}\to \langle \alpha_{0}\rangle ,\\ \alpha_{1}\to \langle \alpha_{1}\rangle \end{array}}{\lim}D)$ in the nonlinear theory. In turn, deficient drag in the adjoint flight would have yielded negative propulsion efficiency at slow flapping rates. In choosing (5.5), we have avoided this outcome, but there are pros and cons for each one of these two definitions.

### 5.3 Level flight

In straight-and-level-constant-speed flight—it will be referred to as ‘SL flight’ below—the average lift counterbalances the weight,

5.17 $$\langle L\rangle =\frac{mg}{\rho Sv^{2}},$$

whereas the average proper thrust counterbalances the drag in the adjoint flight,

5.18 $$\langle T^{\prime }\rangle =\langle {\dot{W}}_{\mathrm{mg}}^{\prime }\rangle =\eta \langle \dot{W}\rangle =\bar{D}=D_{0}+{\bar{D}}_{i};$$

see (5.4), (5.1) and (5.3). The last condition could have been replaced by balancing the energy spent during the flapping cycle with the energy dissipated by drag,

5.19 $$\langle \dot{W}\rangle =\langle D\rangle =D_{0}+\langle D_{i}\rangle ,$$

or by balancing (cancelling) the average proper excess thrust—see (5.2) and (4.22). The following derivations are based on (5.18).

Common measure of cruising performance is the cost of locomotion—in the present context, it is the mechanical energy required per distance flown,

5.20 $$\langle E_{s}^{*}\rangle =\rho v^{2}S\langle \dot{W}\rangle .$$

In view of (5.18) and (5.17), it is simply

5.21 $$\langle E_{s}^{*}\rangle =\frac{mg}{\eta }\frac{\bar{D}}{\bar{L}};$$

recall that $\langle L\rangle =\bar{L}$ by (5.6). It justifies the interpretation of *η*, as defined in (5.4), as ‘efficiency’. Apparently, the lowest cost of locomotion is obtained when the drag-to-lift ratio in the adjoint non-flapping flight, $\bar{D}/\bar{L}$, is minimal (we will return to this point below).

Combination of (5.18), (5.16) and (5.15) yields the variance of the flapping rate in SL flight:

5.22 $$\langle {\dot{\phi }}^{2}\rangle =\frac{\pi }{16}\frac{\bar{D}}{\eta AK_{1}(1-\varepsilon )}=\frac{H_{1}(A,\varepsilon )}{1-\varepsilon }\bar{D},$$

where

**Figure 6.**
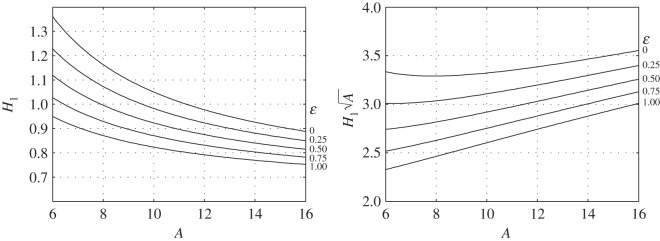
Functions *H*_1_(*A*, *ε*) and $H_{1}(A,\varepsilon )\sqrt{A}$ for several values of *ε*.

5.23 $$H_{1}(A,\varepsilon )=\frac{\pi }{16AK_{1}\eta }=\frac{\pi }{16A(K_{1}-K_{2}(1-\varepsilon ))}$$

is shown in figure 6*a*. In turn, $\left\langle {\dot{\phi }}^{2}\right\rangle$is related to flapping amplitude (*ϕ*_0_) and angular frequency (*ω*) by

5.24 $$\langle {\dot{\phi }}^{2}\rangle =k_{\phi }\omega^{2}\phi_{0}^{2},$$

where *k*_*ϕ*_ is a certain parameter depending on the flapping pattern. It equals 1/2 when *ϕ* varies with time as a sine, and 1/(*π*^2^*τ*_*d*_(1−*τ*_*d*_)) when it varies as a ‘saw-tooth’ with *τ*_*d*_ being the relative part of the down stroke in the flapping period.

Combination of (5.22) and (5.24) furnishes the flapping frequency in SL flight:

5.25 $$\omega^{2}=\frac{H_{1}(A,\varepsilon )}{k_{\phi }}\frac{\bar{D}}{\phi_{0}^{2}(1-\varepsilon )};$$

its dimensional variant is

**Figure 7.**
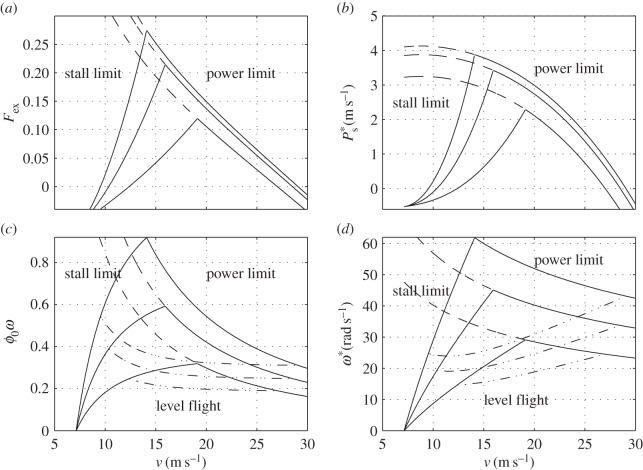
(*a*) Specific excess thrust, (*b*) specific excess power, (*c*) reduced flapping rate amplitude and (*d*) flapping frequency for the hypothetical bird specified in appendix G. The three sets of lines in each figure correspond to *ε*=0 (inner set), *ε*=0.5 (middle set) and *ε*=0.7 (outer set). Dashed lines mark the power limit beyond stall. Dash-dotted lines in panels (*c*,*d*) mark the flapping frequency in SL flight.

5.26 $$\omega^{*2}=\frac{H_{1}(A,\varepsilon )}{k_{\phi }}\frac{mg}{\rho Ss^{2}}\frac{1}{\phi_{0}^{2}(1-\varepsilon )}\frac{\bar{D}}{\bar{L}}$$

by (5.17), (5.15) and (5.6). An example is shown in figure 7*c*,*d*. Referring to figure 7*c*, one may note that the reduced flapping rate *ϕ*_0_*ω* needed to sustain SL flight is a few tenths. It furnishes, by way of example, an *a posteriori* verification of the small angles assumption made in §4.1.

It follows from (5.26) that the dimensional (‘real’) flapping frequency changes with the square-root of the drag-to-lift ratio in the adjoint non-flapping flight, $\bar{D}/\bar{L}$, and because

5.27 $$\frac{\bar{D}}{\bar{L}}=\frac{D_{0}}{\bar{L}}+\frac{\bar{L}}{\pi A}=\frac{\rho SD_{0}}{mg}v^{2}+\frac{mg}{\pi \rho SA}\frac{1}{v^{2}}$$

by (5.3), (5.6), (5.7), (5.12), (5.14) and (5.17), it changes with the flight speed *v* as $\omega^{*}=\sqrt{C_{1}v^{2}+C_{2}/v^{2}},$ where *C*_1_ and *C*_2_ are certain constants. Equation (3) in reference [9] is the Taylor series of this equation. Different behaviour of the flapping frequency with airspeed will imply that the twist parameter *ε* and/or the flapping amplitude *ϕ*_0_ and/or the flapping pattern *k*_*ϕ*_ change in flight.

The drag-to-lift ratio $\bar{D}/\bar{L}$ has a minimum $2\sqrt{D_{0}/\pi A}$ at $v=\sqrt{mg/\rho S}/\sqrt[{4}]{\pi AD_{0}}$; the existence of this (shallow) minimum is manifested in the left-hand side of the flapping frequency curves in figure 7*d* (at 11 m s^−1^).^3^ Assuming that a bird cruises where the cost of locomotion is minimal, the drag-to-lift ratio at cruise should not significantly differ from its minimal value. The flapping frequency at cruise is, therefore,

5.28 $$\omega_{c}^{*}=\frac{D_{0}^{1/4}}{\phi_{0}{\left(1-\varepsilon \right)}^{1/2}}{\left(\frac{mg}{\rho s^{4}}\right)}^{1/2}{\left(\frac{H_{1}(A,\varepsilon )\sqrt{A}}{k_{\phi }\sqrt{\pi }}\right)}^{1/2}$$

by (5.26) and by definition of the aspect ratio. In (5.28), $H_{1}(A,\varepsilon )\sqrt{A}$ is only a weak function of aspect ratio and twist (figure 6*b*), whereas *k*_*ϕ*_ is, practically, a universal constant. In fact, with *k*_*ω*_=1.82,

5.29 $$\omega_{c}^{*}\approx \frac{k_{\omega }D_{0}^{1/4}}{\phi_{0}{\left(1-\varepsilon \right)}^{1/2}}{\left(\frac{mg}{\rho s^{4}}\right)}^{1/2}$$

approximates (5.28) to within 9% for every *A* in (6,16) and every *ε* in (0.1, 0.9). Equation (5.29) can be seen as a rational variant of equations (10) in [10] and (9) in [11].

**Figure 8.**
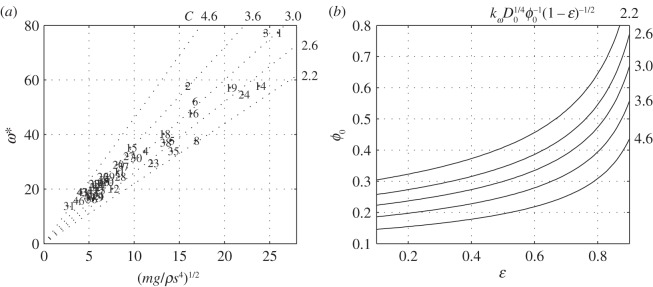
(*a*) Observed flapping frequency and (*b*) estimated flapping amplitude at cruise. Forty-six numbered markers in the left figure represent 46 species compiled in appendix D of part 2. The slope *C* of the dotted lines in the left figure is shown to the right of each line. The lines in the left figure show combinations of *ϕ*_0_ and *ε* for which $k_{\omega }D_{0}^{1/4}\;\phi_{0}^{-1}{\left(1-\varepsilon \right)}^{-1/2}=C$, with *D*_0_=0.015 and *k*_*ω*_=1.81.

Morphological data of a few species of birds and observations of their respective flapping frequencies at cruise have been compiled in appendix D of part 2. It is shown in figure 8*a* against $\sqrt{mg/\rho s^{4}}$. For the 46 species represented in figure 8*a*, the ratio (say, *C*) of the two is bounded to the interval (2.2,4.6). Accepting (5.29), this ratio equals $k_{\omega }D_{0}^{1/4}\;\phi_{0}^{-1}{\left(1-\varepsilon \right)}^{-1/2}$. Because *k*_*ω*_ can change only within ±9% from its nominal value, and because doubling *D*_0_ changes $D_{0}^{1/4}$ by less than 20%, the observed range of *C* can be explained only by differences in the flapping amplitude and the twist parameter. Combinations of *ϕ*_0_ and *ε* that yield relevant values of *C* are shown in figure 8*b*. We found no reliable data on the twist parameter. Nonetheless, based on a few available observations [12–15], *ϕ*_0_ is of the order of 0.5 rad. It implies that the twist parameter *ε* for all the species represented in figure 8 exceeds 0.5.

### 5.4 Specific excess power

Common measures of flight performance are the specific excess thrust, *F*_ex_, and the specific excess power, P*_s_ [6]. Specific excess thrust is defined as the ratio between the proper excess thrust (the difference between the maximal available thrust and drag) and weight. Specific excess power is defined as the product of the specific excess thrust and airspeed

5.30 $$P_{s}^{*}=vF_{\mathrm{ex}}.$$

At given flight conditions, *F*_ex_ can be interpreted as the level acceleration (in ‘*g*’ units) and hence is equivalent to the maximal sustained climb angle (if it is sufficiently small); P*_s_ can be interpreted as the rate of change of the energy altitude and hence is equivalent to the maximal sustained climb rate. Formally,

5.31 $$F_{\mathrm{ex}}=\frac{\eta P_{\max}-\bar{D}}{\bar{L}}=\frac{P_{\max}-\langle D\rangle }{\bar{L}},$$

where $P_{\max}$ is the maximal available power. When *F*_ex_=0, equation (5.31) becomes a variant of (5.18) or a variant of (5.19).

In flapping flight, the maximal available power can be limited either by the maximal sustained power, $P_{a}^{*}$, or by stall. In the first case,

5.32 $$P_{\max}=\frac{P_{a}^{*}}{\rho Sv^{3}},$$

and, consequently,

5.33 $$F_{\mathrm{ex}}=\frac{\eta P_{a}^{*}}{mgv}-\frac{\bar{D}}{\bar{L}}.$$

Because for a constant *ε*, *η* is independent of airspeed (see (5.15)), equations (5.33) and (5.30) are the same as for a propeller-driven fixed-wing aeroplane. An example is shown in figure 7*a*,*b*. Both *F*_ex_ and $P_{s}^{*}$ diminish with airspeed; they vanish at the maximal speed that can be obtained in horizontal flight (in this example, 27–29 m s^−1^, depending on the twist parameter).

The stall limit has no analogy with fixed-wing aeroplanes. It was shown in §4.4 that the maximal angle of attack along the span is

5.34 $$\alpha_{c}=\frac{\bar{L}}{2\pi }+K_{3}\dot{\phi }(1-\varepsilon );$$

this equation follows (4.35) by (5.11), (5.12), (5.14) and (5.6). If *α*_*c*_ is not to exceed the onset of stall at $\alpha_{\max}=L_{\max}/2\pi$, $\dot{\phi }$ should be limited from above by

5.35 $${\dot{\phi }}_{\max}=\frac{L_{\max}-\bar{L}}{2\pi K_{3}(1-\varepsilon )}.$$

This limit on $\dot{\phi }$ can be translated into a limit $k_{\dot{\phi }}{\dot{\phi }}_{\max}^{2}$ on the variance $\left\langle {\dot{\phi }}^{2}\right\rangle$of the flapping rate ($k_{\dot{\phi }}$ is akin to *k*_*ϕ*_ in (5.24)—both equal 1/2 if the flapping is harmonic); and, in turn, into a limit

5.36 $$P_{\max}=\underset{\langle {\dot{\phi }}^{2}\rangle \to k_{\dot{\phi }}{\dot{\phi }}_{\max}^{2}}{\lim}\dot{W}=\frac{16}{\pi }\frac{AK_{1}k_{\dot{\phi }}}{\left(1-\varepsilon \right)}{\left(\frac{L_{\max}-\bar{L}}{2\pi K_{3}}\right)}^{2},$$

on the maximal power that can be supplied to the wings without stalling a part of them (see (5.16)). Consequently,

5.37 $$F_{\mathrm{ex}}=\frac{4k_{\dot{\phi }}}{\pi^{3}}\frac{A(K_{1}-\left(1-\varepsilon \right)K_{2})}{\left(1-\varepsilon \right)K_{3}^{2}}\frac{{\left(L_{\max}-\bar{L}\right)}^{2}}{\bar{L}}-\frac{\bar{D}}{\bar{L}}$$

by (5.31) and (5.15). It manifests a rapidly increasing function of airspeed (figure 7*a*).

Reiterating, a bird is limited by stall at low speed, and by power at high speed. The best climb rate and the best climb angle are achieved where both limitations meet. Increasing the twist decreases the minimal flight speed (see (5.37)), and increases both the maximal flight speed and the maximal climb rate (see (5.15) and (5.33))). Increasing the twist above *ε*=0.5 has practically no effect on the specific power—and hence on the climb rate—but has a dramatic effect on the reduced frequency, especially at low speed. Increasing the reduced frequency eventually makes the velocity of the wing owing to flapping comparable to the flight velocity (figure 7*c*), and it is here where the present theory fails. Low-speed phases of flight require different analysis (e.g. [16–18]). Nonetheless, the stall limit does have significance—it marks the transition between forward and hovering flight. Flight beyond the stall limit is possible only by tilting the flapping plane so as to direct most of the thrust upwards [19].

## 6. Free flight

### 6.1 Formulation

We release now the degrees of freedom of the bird's body (that is, take the bird off the sting) and seek the resulting effect on performance. The degrees of freedom involve heave and pitch; the relationship between the two depends on the active control strategy adopted by the bird. The two most obvious strategies, keeping the angle-of-attack (*α*_0_) constant, and keeping the pitch angle (*τ*) constant, are addressed in §§6.2 and 6.3 below. For the sake of simplicity, we assume that the twist at the shoulder does not change with time—that is, we adopt (5.9); we also assume that the mass of the wings is negligible when compared with the mass of the body.

Under these assumptions, the heave is governed by the equation

6.1 $$m\ddot{h}=\rho Ss(L-\langle L\rangle ),$$

where *L* is given by (4.24), and $\langle L\rangle =\bar{L}$ satisfies (5.17). With (4.24), (5.12), (5.9), (4.4) and (A 7), equation (6.1) can be recast as

6.2 $$\ddot{h}=s_{1}\left(\alpha_{0}-\langle \alpha_{0}\rangle +\frac{4}{3\pi }\alpha_{1}\right),$$

where

6.3 $$s_{1}=\pi AA_{1}\frac{\rho Ss}{m}$$

is a parameter. It can be interpreted as the ratio of the lift slope coefficient, *L*_,*α*_=*πAA*_1_ (see (4.24)), and the reduced mass, *m*/*ρ*Ss. For most of the species compiled in appendix D of part 2, *s*_1_ varies in the interval (0.1, 1).

### 6.2 Constant angle of attack

Flying at a constant angle of attack implies that

6.4 $$\alpha_{0}=\langle \alpha_{0}\rangle$$

throughout the flapping cycle. Consequently,

6.5 $$\ddot{h}=s_{1}\frac{4}{3\pi }\alpha_{1}=s_{1}\frac{4}{3\pi }(1-\varepsilon )\dot{\phi }$$

by (5.11) and (6.2), from which (upon multiplying it by $\dot{h}$ and averaging over a single period):

6.6 $$\langle \alpha_{1}\dot{h}\rangle =\frac{3\pi }{4s_{1}}\langle \dot{h}\ddot{h}\rangle =0.$$

Introducing (6.4), (6.6) and (5.11) in (5.8) and (4.32), recovers (5.15) and (5.16). In other words, if the angle of attack is kept constant during flight, both the propulsion efficiency and the input power remain unaffected by the motion of the bird's body. A wind-tunnel experiment with a bird tied to a sting should yield the same results as if the bird was in free flight.

### 6.3 Constant pitch

Flying at constant pitch angle implies $\dot{\tau }=0$. Introducing it, together with (5.9), (5.12), (4.4) and (A 7), in (6.2) yields

6.7 $$\ddot{h}+s_{1}\dot{h}=\frac{4}{3\pi }s_{1}\alpha_{1}.$$

Some progress can be made before actually solving (6.7). In fact, multiplying it by $\dot{h}$, and averaging over a single period, yields $\left\langle {\dot{h}}^{2}\rangle =(4/3\pi )\langle \alpha_{1}\dot{h}\right\rangle$. At the same time, because ${\dot{\alpha }}_{g0}=0$ by (5.9) and $\dot{\tau }=0$ by assumption, $\left\langle \alpha_{0}^{2}\rangle -{\left\langle \alpha_{0}\right\rangle }^{2}=\langle {\dot{h}}^{2}\rangle =-\langle \alpha_{0}\dot{h}\right\rangle$ and $\left\langle \alpha_{1}\alpha_{0}\rangle =-\langle \alpha_{1}\dot{h}\right\rangle$ by (4.4). Moreover, $\left\langle \alpha_{1}\dot{\phi }\rangle =\langle \alpha_{1}^{2}\rangle /(1-\varepsilon \right)$ and $\left\langle \alpha_{0}\dot{\phi }\rangle =\langle \alpha_{0}\alpha_{1}\rangle /(1-\varepsilon \right)$ by (5.11). Substituting these, together with (4.31) and (A 7), in (5.8) and (4.32) furnishes

6.8 $$\eta =1-(1-\varepsilon )A_{1}\frac{k_{2}}{k_{1}}K_{\eta }$$

and

6.9 $$\langle \dot{W}\rangle =\frac{16}{9\pi }\frac{AA_{1}k_{1}}{1-\varepsilon }\langle \alpha_{1}^{2}\rangle K_{\dot{W}},$$

where

6.10 $$K_{\eta }=\left(1-\frac{9\pi^{2}}{16k_{2}}\frac{\left\langle {\dot{h}}^{2}\right\rangle }{\left\langle \alpha_{1}^{2}\right\rangle }\right){\left(1-\frac{9\pi^{2}}{16k_{1}}\frac{\left\langle {\dot{h}}^{2}\right\rangle }{\left\langle \alpha_{1}^{2}\right\rangle }\right)}^{-1}$$

and

6.11 $$K_{\dot{W}}=1-\frac{9\pi^{2}}{16k_{1}}\frac{\left\langle {\dot{h}}^{2}\right\rangle }{\left\langle \alpha_{1}^{2}\right\rangle }$$

are the respective correction factors. Because *k*_1_>0 and *k*_2_>*k*_1_>0 by (4.30), $K_{\dot{W}}<1$ and *K*_*η*_>1. Consequently, $\left\langle {\dot{h}}^{2}\right\rangle$ has a detrimental effect on the propulsion efficiency and on the input power. When $\left\langle {\dot{h}}^{2}\right\rangle$ vanishes, (6.8) and (6.9) recover (5.15) and (5.16), respectively.

If $\phi =\phi_{0}\cos\omega$t,

6.12 $$\left\langle {\dot{h}}^{2}\rangle =\frac{s_{1}^{2}}{s_{1}^{2}+\omega^{2}}\frac{16}{9\pi^{2}}\langle \alpha_{1}^{2}\right\rangle$$

by (6.2), and, concurrently,

6.13 $$\langle \alpha_{1}^{2}\rangle ={\left(1-\varepsilon \right)}^{2}\langle {\dot{\phi }}^{2}\rangle =\frac{1}{2}\phi_{0}^{2}\omega^{2}{\left(1-\varepsilon \right)}^{2}$$

by (5.11). Combination of (6.9), (6.11), (6.12) and (6.13) furnishes a quadratic equation for (*ω*/*s*_1_)^2^:

6.14 $$k_{1}{\left(\frac{\omega }{s_{1}}\right)}^{4}+\left(k_{1}-1-d\right){\left(\frac{\omega }{s_{1}}\right)}^{2}-d=0,$$

where

6.15 $$d=\frac{9\pi \langle \dot{W}\rangle }{8AA_{1}s_{1}^{2}\phi_{0}^{2}(1-\varepsilon )}=\frac{9\langle \dot{W}\rangle }{\pi \phi_{0}^{2}{\left(2AA_{1}\right)}^{3}(1-\varepsilon )}{\left(\frac{m}{\rho Ss}\right)}^{2}$$

is a parameter; the right-hand side of (6.15) follows by (6.3). The relevant solution of (6.14) is

6.16 $${\left(\frac{\omega }{s_{1}}\right)}^{2}=-\frac{k_{1}-1-d}{2k_{1}}+\sqrt{{\left(\frac{k_{1}-1-d}{2k_{1}}\right)}^{2}+\frac{d}{k_{1}}}.$$

**Figure 9.**
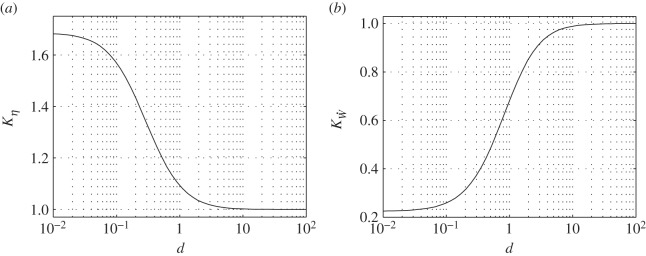
Free heave corrections for (*a*) efficiency and (*b*) power.

Substituting it in (6.12), and the result in (6.10) and (6.11), yields *K*_*η*_ and $K_{\dot{W}}$ as functions of *d*. They are shown in figure 9.

The limit d→∞ corresponds to m→∞ by (6.15), and hence $\underset{d\to \infty }{\lim}\dot{h}=0$ by (6.2). Indeed, in this case, both correction factors tend to unity. For the hypothetical bird of appendix G, $d\approx 300\langle \dot{W}\rangle$. Because $\left\langle \dot{W}\right\rangle$ is comparable to the (reduced) drag, a few hundredths, *d* turns out to be of the order of 10. It implies negligible effects of free heave on the propulsion efficiency and the input power. Nonetheless, a combination of small twist, low wing loading and large flapping amplitude may reduce *d* by an order of magnitude. Should this be the case, flying with constant pitch may still have an imperceptible effect on efficiency, but may have a profound effect on the input power.

## 7. Concluding remarks

An aerodynamic model based on the lifting line theory seems to provide a good balance between accuracy (§§4.5 and 5.2) and simplicity (§§4.3 and 4.4). Its main limitation is the low-speed phases of flight, where the velocity of the wing owing to flapping is comparable to (or exceeds) the flight velocity. Better theories in this respect (e.g. [16]) are too complicated to be used effectively in analysis of the type that was carried through here, and will be carried through in part 2.

Wing twist was identified as the central parameter affecting performance (§§5.1 and 5.4). It improves efficiency, and hence improves both the maximal rate of climb and the maximal level speed. It also reduces the angle of attack at the wing's tip, and hence allows the bird to fly slower. For 46 species of birds analysed in this study, the twist parameter at cruise should have been more than 0.5; that is, the twist at the tip should have been larger than half the angle of attack induced by flapping.

The flapping frequency required to sustain a level-constant-speed flight is proportional to the square root of the drag-to-lift ratio in the adjoint flight. It is minimal when the drag-to-lift ratio is minimal—in fact, where the cost of locomotion is minimal.

The following list summarizes the most important of the odd 100 equations of the paper. Aerodynamic loads: (4.24)–(4.31); angle of attack: (4.35); proper thrust: (5.1); adjoint flight: (5.5); propulsion efficiency: (5.4), (5.15), (6.8); power: (4.32), (5.16), (6.9); flapping frequency: (5.26), (5.28), (5.29); cost of locomotion: (5.21); specific power and specific excess thrust: (5.30) and (5.31).

## Supplementary Material

Corroboration of the code

## Supplementary Material

Corroboration of the theory

## Figures and Tables

**Figure 10.**
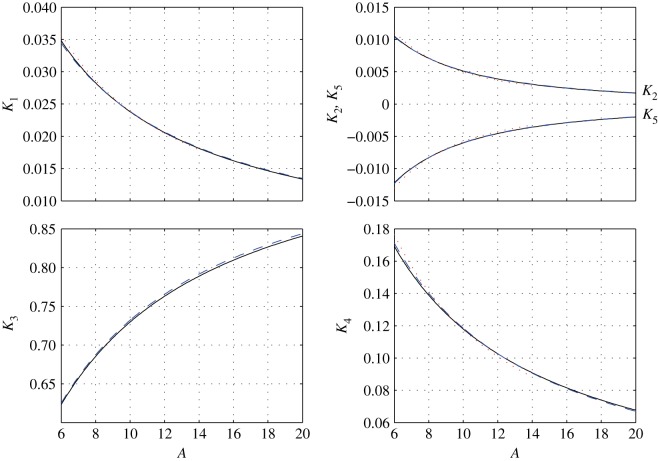
Functions *K*_1_, …, *K*_5_ and their approximations. Infinite sums are shown with solid lines; approximations (B 1)–(B 3) are shown with dashed lines; approximations (4.30)–(4.31) are shown with dotted lines.

**Figure 11.**
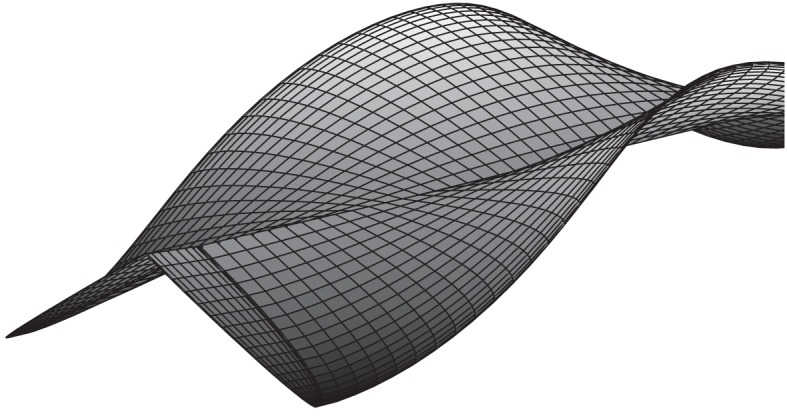
A prescribed wake. The generating wing is visible on the left.

## Footnotes

1 In all these cases, the flapping was harmonic, with $\phi (t)=\phi_{0}\cos\omega t$.

2 *ε* will be formally introduced in (5.10).

3 By differentiating (5.27) with respect to $\bar{L}$, and equating the result to zero, one will find that $\bar{D}/\bar{L}$ has a minimum at $\bar{L}=\sqrt{\pi AD_{0}}$. At this lift, $\bar{D}/\bar{L}=2\sqrt{D_{0}/\pi A}$, $\bar{D}=(\bar{D}/\bar{L})\bar{L}=2D_{0}$, and the respective flight velocity follows by (5.17) and (5.6).

## Data accessibility

This paper uses no external data. Two files have been uploaded with this article as the supplementary material. One file contains 21 cases comparing the aerodynamic theory developed herein with numerical simulations based on the vortex lattice method. The other file contains a verification of the numerical code that was used to generate these simulations.

## Appendix A. Solution of (4.7)

Substituting (4.1), (4.8) and (4.9) in (4.7) yields

A 1 $$\sum_{n=1,3,\ldots }^{\infty }\;\;a_{n}\sin n\theta \left(1+\frac{na}{\pi A}\right)=\frac{a\alpha }{\pi A}\sin\theta \;\mathrm{for}\;\mathrm{each}\;\theta \in (0,\pi ),$$

where *a*=2*π*, and the expression on the left-hand side follows because

A 2 $$--\int_{0}^{\pi }\frac{\cos n\theta^{\prime }d\theta^{\prime }}{\cos\theta^{\prime }-\cos\theta }=\pi \frac{\sin n\theta }{\sin\theta }$$

[7, p. 469]. Integrating (A 1) with sin *nθ* over (0, *π*) furnishes *a*_1_, *a*_3_, … as quadratures,

A 3 $$a_{n}=\frac{2A_{n}}{\pi }\int_{0}^{\pi }\alpha \sin\theta \sin n\theta d\theta ,$$

where *A*_*n*_ is given by (4.11). When *α* is given by (4.3), (A 3) yields (4.10). The standard integrals,

A 4 $$I_{mn}=\int_{\pi /2}^{\pi }\sin n\theta \sin m\theta \cos\theta d\theta ,$$

appearing in (4.10), can be evaluated to obtain

A 5 $$I_{mn}=\frac{1}{2}\left(\frac{{\left(-1\right)}^{n-m/2}}{{\left(n-m\right)}^{2}-1}-\frac{{\left(-1\right)}^{{\;}^{n+m/2}}}{{\left(n+m\right)}^{2}-1}\right)$$

when the sum *n*+*m* is even, and

A 6 $$I_{mn}=\frac{\pi }{8}(\delta_{m,n+1}+\delta_{m+1,n})$$

when it is odd; useful particular cases are

A 7 $$I_{11}=-\frac{1}{3}\;\mathrm{and}\;I_{13}=-\frac{1}{5}.$$

## Appendix B. Functions *K*_1_, …, *K*_5_

Functions *K*_1_, …, *K*_5_ have been defined in (4.28), (4.29) and (4.36) as infinite sums involving *A*_*n*_ from (4.11), and *I*_*mn*_ from (A 4). They can be furnished with simple Padé approximations:

B 1 $$\begin{array}{rlrl} 2K_{1} & \approx \frac{0.31}{A+3}, & \;K_{2} & \approx \frac{1.02}{\left(A+1)(A+8\right)}, \end{array}$$

B 2 $$\begin{array}{rl} K_{3} & \approx \frac{A+0.25}{A+4}, \end{array}$$

B 3 $$\begin{array}{rlrl} K_{4} & \approx \frac{1.54}{A+3}, & \;K_{5} & \approx -\frac{1.12}{\left(A+1)(A+7\right)}, \end{array}$$

which are practically indistinguishable from the 64-terms sums shown in figure 10.

## Appendix C. Equivalence between (4.33) and (4.34)

Because the coefficients of *α*_0_ in (4.33) and (4.34) match by (4.11), it suffices to demonstrate that coefficients of *α*_1_ match as well, or, to the same end, that

C 1 $$\sin\theta |\cos\theta |=-\frac{2A}{\pi }\sum_{n=1,3,5,\ldots }^{\infty }\;\;A_{n}I_{1n}\sin n\theta \left(1+2n/A\right).$$

Multiplying this equation by sin *mθ* and integrating between 0 and ***π*** yields −(1−(−1)^*m*^)*I*_1*m*_ in the left-hand side, by (D 1), and, indeed, −*AA*_*m*_*I*_1*m*_(1+2*m*/*A*)=−2*I*_1*m*_ in the right-hand side, by (5.11). Because the equivalence holds for any *m*, the proposition follows.

## Appendix D. Extremum of the angle of attack at the wing tip

In order to demonstrate that the angle of attack is maximal at the wing tips, it suffices to demonstrate that the derivative of (4.34) with respect to *θ*,

D 1 $$\sum_{n=1,3,5,\ldots }^{\infty }\;\;A_{n}I_{1n}\frac{n\sin\theta \cos n\theta -\cos\theta \sin n\theta }{\sin^{2}\theta },$$

vanishes at *θ*=0 and *θ*=*π*. Noting the expansions

D 2 $$\sin n\theta =n\sin\theta \left(1-\frac{\sin^{2}\theta (n^{2}-1^{2})}{3!}+\frac{\sin^{4}\theta (n^{2}-1^{2})(n^{2}-3^{2})}{5!}+\cdots \right)$$

and

D 3 $$\cos n\theta =\cos\theta \left(1-\frac{\sin^{2}\theta (n^{2}-1^{2})}{2!}+\frac{\sin^{4}\theta (n^{2}-1^{2})(n^{2}-3^{2})}{4!}+\cdots \right),$$

applicable to any odd *n* ([20], art. 3.172), this sum in (D 1) equals

D 4 $$\sum_{n=1,3,5,\ldots }^{\infty }\;\;A_{n}I_{1n}n\sin2\theta \left(-\frac{n^{2}-1^{2}}{3!}+2\sin^{2}\theta \frac{\left(n^{2}-1^{2})(n^{2}-3^{2}\right)}{5!}+\cdots \right).$$

It indeed vanishes at *θ*=0 and *θ*=*π*.

## Appendix E. Numerical methods

One of the classical methods to obtain aerodynamic loads acting on a moving surface is the vortex lattice method [21]. The particular implementation of this method for this study was based on vortex ring elements; it followed the paradigm described in [21] to a point. Wake rollup was inhibited, but the wake traced the path of the trailing edge (figure 11). The pressure distribution on the wing was computed using the working formulae of [21]; the integral loads (*L*, *T*, *M*_*x*_, *M*_*y*_) followed by quadratures, again, exploiting the formulae of [21]. The power was computed from the pressure distribution and the velocity of the wing points relative to C. The propulsion efficiency was calculated using (5.4). ${\bar{D}}_{i}$, needed to this end, was calculated by setting the wing at the same angle of attack, and adjusting the dihedral angle until the average lift was matched. The same result could have been obtained by flapping the wings with a very low frequency (say, 0.01) and the same amplitude. Practically, however, the same result could have been obtained by setting the wing fully spread—the difference would have been only a couple per cent.

The code was corroborated by comparison with third-order asymptotic lifting line theory [22] and two-dimensional unsteady thin wing theory [23,24]; details can be found in the electronic supplementary material.

## Appendix F. Numerical simulations

In all simulations, the wing had an elliptical plan-form with straight quarter-chord line and linear twist. Flapping was harmonic, $\phi (t)=\phi_{0}\cos\omega t$, with (reduced) frequency *ω* and amplitude *ϕ*_0_. There was no pitch and no heave. There were 19 spanwise and 19 chordwise cells on a single (right) wing. The simulations continued for 150 steps; the time step was adjusted to obtain 50 steps per period. Other parameters are specified in table 2. The cases shown in figures 3 and 4 have been boldfaced.

**Table 2.** List of numerical simulations.

*A*	*ϕ*_0_ (°)	*ω*	*α*_*g*,0_(°)	*ε*
7, 10, 14	15	0.8, 1.2	5	0.3, 0.5, 0.7
6, **8**, 12, 16	30	**0.8**, **1.2**, 1.6	5	0.3, **0.5**, 0.7

## Appendix G. Data for performance analysis

A hypothetical mid-sized bird was generated for analysis, resembling Cape petrel (*Daption capense*) in mass and dimensions (table 3). The maximal lift coefficient chosen for this bird probably exceeds the maximal lift coefficient that can be expected at the relevant Reynolds number (which is about 70 000 at 10 m s^−1^), even when corrected for possible delay of stall because of flapping. Increasing the maximal lift coefficient allowed for a clearer demonstration of the minimum of the flapping frequency in level flight. The parasite drag coefficient was chosen on the lower side of what could have been expected based on parasite drag of a typical profile (see appendix D of part 2); this choice served the same purpose of clearer visualization. Maximal power was assumed after [12]; the flapping was harmonic, $\phi (t)=\phi_{0}\cos\omega t$, with (reduced) frequency *ω* and amplitude *ϕ*_0_; there was no pitch and no heave; the air density was standard, *ρ*=1.225 kg m^−3^.

**Table 3.** Parameters of a hypothetical bird.

*M* (kg)	2*s* (m)	2*S* (m^2^)	*A*	*ϕ*_0_ (°)	*ε*	$L_{\max}$	*P**_*a*_ (W)	*D*_0_
0.4	0.8	0.08	8	30	0, 0.5, 0.7	1.6	20	0.015
