# Supplementary material for: Forward flight of birds revisited. Part 1: aerodynamics and performance
Source: R Soc Open Sci. 2014 Oct 15;1(2):140248. doi: 10.1098/rsos.140248 (PMC4448904; doi:10.1098/rsos.140248)
Supplement: Corroboration of the code [file rsos140248supp1.pdf]

Corroboration of the code

Shown here is the comparison between the numerical simulations, the 3rd order asymptotic lifting line theory [1] (figure S1.1), and the 2D unsteady thin wing theory [2, 3] (figure S1.2). Details of the test cases are specified below.

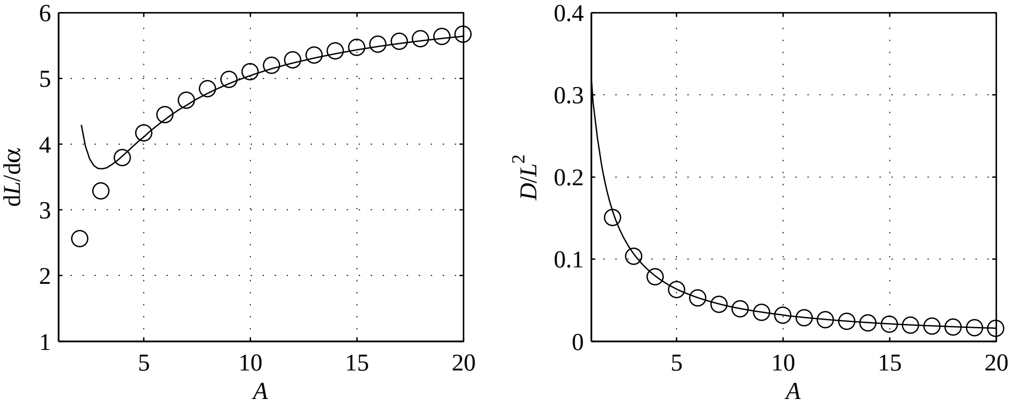

Figure S1.1: Lift slope and induced drag of an elliptic wing. Conditions are those of case 1. 3rd order asymptotic theory for high aspect ratio (equation (N.29) in [1]) is shown with solid lines; numerical simulations are shown with circles. The match is very good, except for $A < 4$, where the asymptotic theory is not supposed to work.

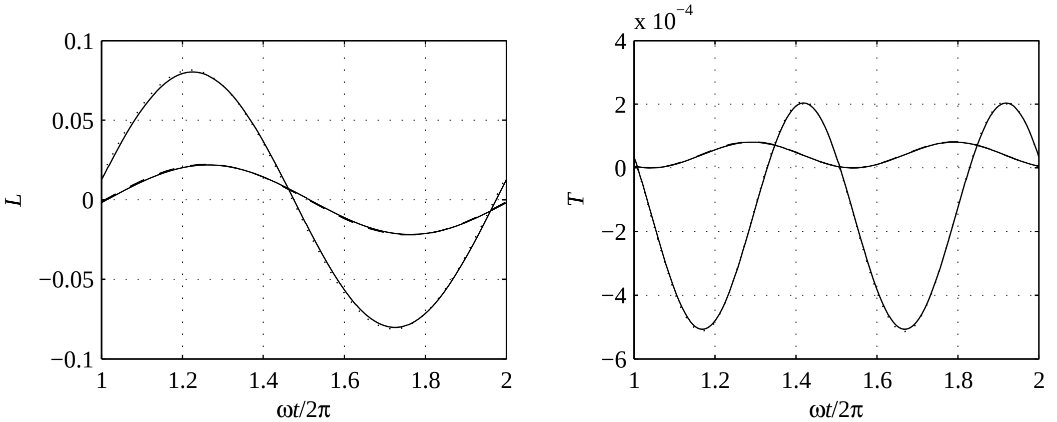

Figure S1.2: Lift and thrust coefficients of a rectangular wing of aspect ratio 100 performing pure pitch (dotted line) and pure heave (dashed line) oscillations. Dimensionless frequency based on semi-chord is 0.25. Conditions are those of cases 2 and 3. The heave velocity and the pitch angle are zero at $t/T = 1$. Analytical solutions of Theodorsen [2] and Garrick [3] are shown with solid lines.

Case 1

The first test case was based on a planar wing with elliptical chord distributions and straight quarter-chord line. The wing was set at 1 degree angle of attack. The number of span-wise (N_s) and chord-wise (N_c) cells on the right wing was 19 and 9, respectively. The aspect ratio varied between 2 and 20 in steps of 1.

Case 2

The second test case was based on a rectangular wing of aspect ratio 100. The wing oscillated harmonically in heave with reduced frequency of 0.25 (based on the semi-chord) and amplitude of 0.01 chords. The number of span-wise (N_s) and chord-wise (N_c) cells on the right wing was 1 and 39, respectively. The reduced time step was 0.125 characteristic times (the ratio of the semi-chord to the flow velocity\); the number of simulation steps was 500.

Case 3

The third case was similar to case 2 with the difference that the wing oscillated in pitch about the quarter-chord point (rather than in heave) with amplitude of 1 deg.

References

[1] Van Dyke M., *Perturbation methods in fluid mechanics*, Parabolic Press, 1975

[2] Bisplinghoff R., Ashley H., Halfman R., *Aeroelasticity*, Dover, 1996, p. 272

[3] Garrick I. E., *Propulsion of a flapping and oscillating airfoil*, NACA Rept. 567, 1936.
