# Supplementary material for: Forward flight of birds revisited. Part 1: aerodynamics and performance
Source: R Soc Open Sci. 2014 Oct 15;1(2):140248. doi: 10.1098/rsos.140248 (PMC4448904; doi:10.1098/rsos.140248)
Supplement: Corroboration of the theory [file rsos140248supp2.pdf]

Corroboration of the theory

This file contains 21 figures showing the comparison between numerical simulations and the lifting-line theory. Each figure shows the reduced lift *L*, thrust *T*, pitching moment M_y, and power $\dot{W}$ as functions of the reduced time. The units of force, moment, power and time are $\rho S v^2$, $\rho S s v^2$, $\rho S v^3$ and s/v, respectively, where ρ, *v*, *S* and *s* are the air density, the flight velocity, the area and the span of a single wing. Dots mark the numerical simulations; solid lines mark theoretical predictions with equations (29), (32), (37) and (27). Wing positions during flapping are shown, schematically, at the bottom of each figure. The motion of the wing is assumed harmonic, with $\phi(t) = \phi_0 \cos \omega t$. Numerical simulation used 19 span-wise and 19 chord-wise cells (on one wing), 100 wake cells and time step of $2\pi/50\omega$. The cases shown in the figures differ in flapping amplitude ϕ_0, flapping frequency ω, aspect ratio A, and twist parameter ε. In all the cases, the wing had an elliptical plan-form with straight quarter-chord line.

figure	A	ϕ_0 (deg)	ε	ω
S2.1	6	30	0.3	0.8,1.2,1.6
S2.2	6	30	0.5	0.8,1.2,1.6
S2.3	6	30	0.7	0.8,1.2,1.6
S2.4	7	15	0.3	0.8,1.2
S2.5	7	15	0.5	0.8,1.2
S2.6	7	15	0.7	0.8,1.2
S2.7	8	30	0.3	0.8,1.2,1.6
S2.8	8	30	0.5	0.8,1.2,1.6
S2.9	8	30	0.7	0.8,1.2,1.6
S2.10	10	15	0.3	0.8,1.2
S2.11	10	15	0.5	0.8,1.2
S2.12	10	15	0.7	0.8,1.2
S2.13	12	30	0.3	0.8,1.2,1.6
S2.14	12	30	0.5	0.8,1.2,1.6
S2.15	12	30	0.7	0.8,1.2,1.6
S2.16	14	15	0.3	0.8,1.2
S2.17	14	15	0.5	0.8,1.2
S2.18	14	15	0.7	0.8,1.2
S2.19	16	30	0.3	0.8,1.2,1.6
S2.20	16	30	0.5	0.8,1.2,1.6
S2.21	16	30	0.7	0.8,1.2,1.6

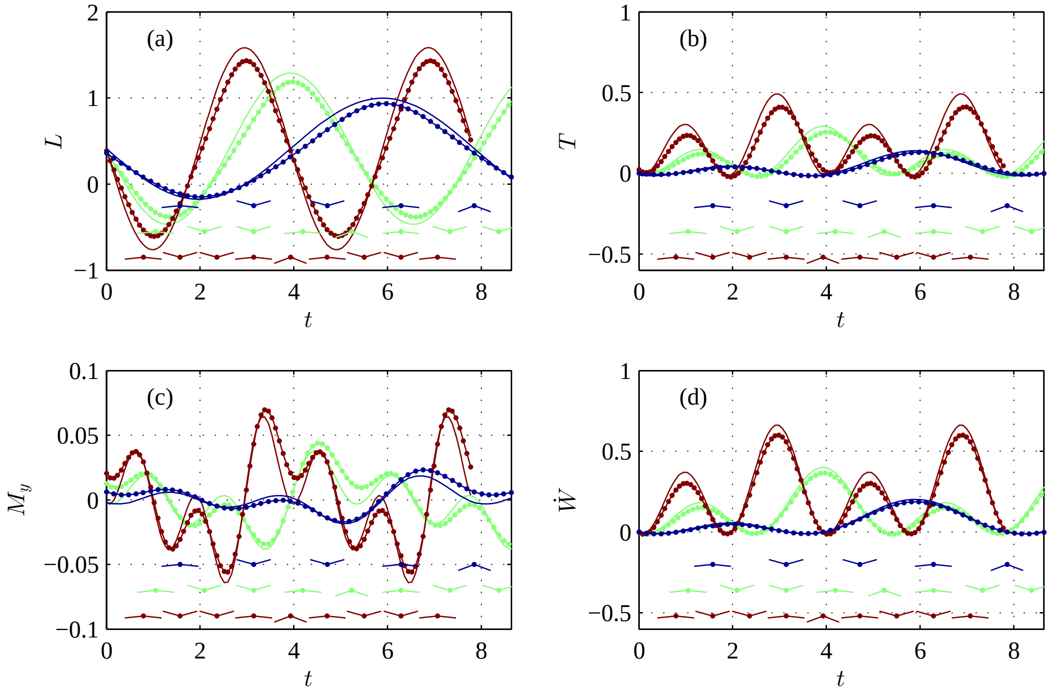

Figure S2.1: $A=6$, $\phi_0=30$, $\varepsilon=0.3$. $\omega=0.8$ (blue) $\omega=1.2$ (green) and $\omega=1.6$ (red).

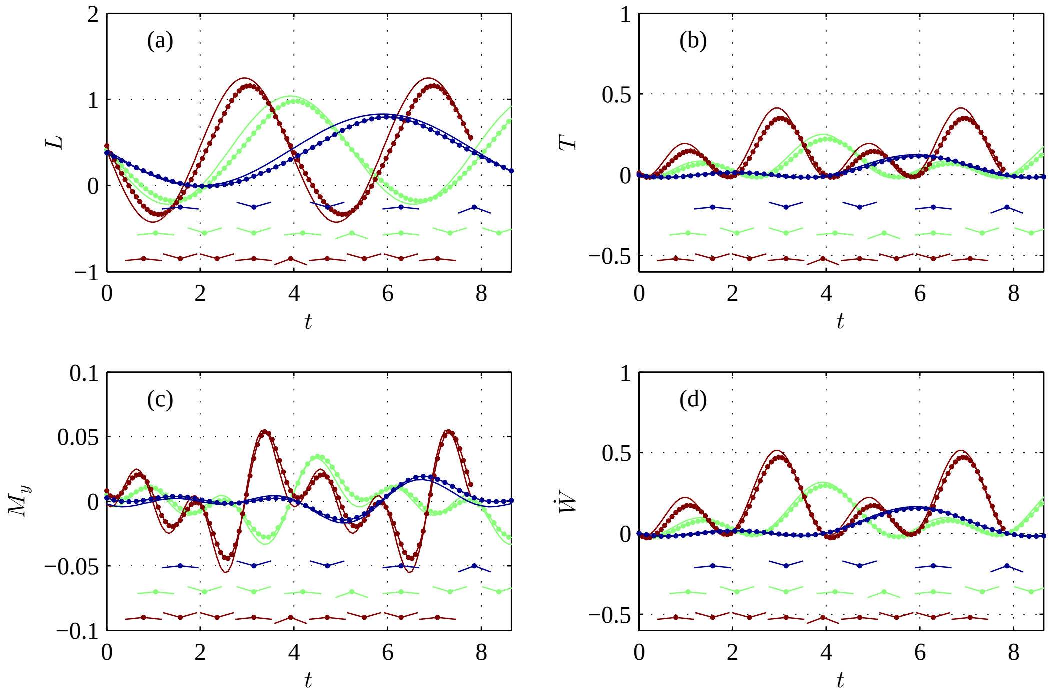

Figure S2.2: $A=6$, $\phi_0=30$, $\varepsilon=0.5$. $\omega=0.8$ (blue) $\omega=1.2$ (green) and $\omega=1.6$ (red).

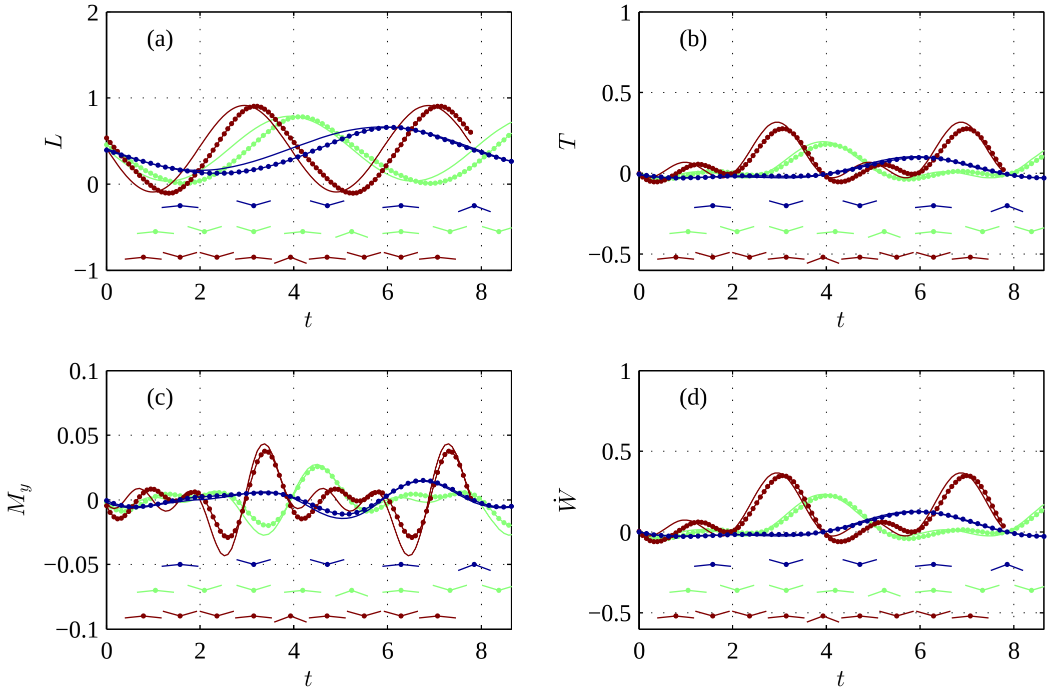

Figure S2.3: $A = 6$, $\phi_0 = 30$, $\varepsilon = 0.7$. $\omega = 0.8$ (blue) $\omega = 1.2$ (green) and $\omega = 1.6$ (red).

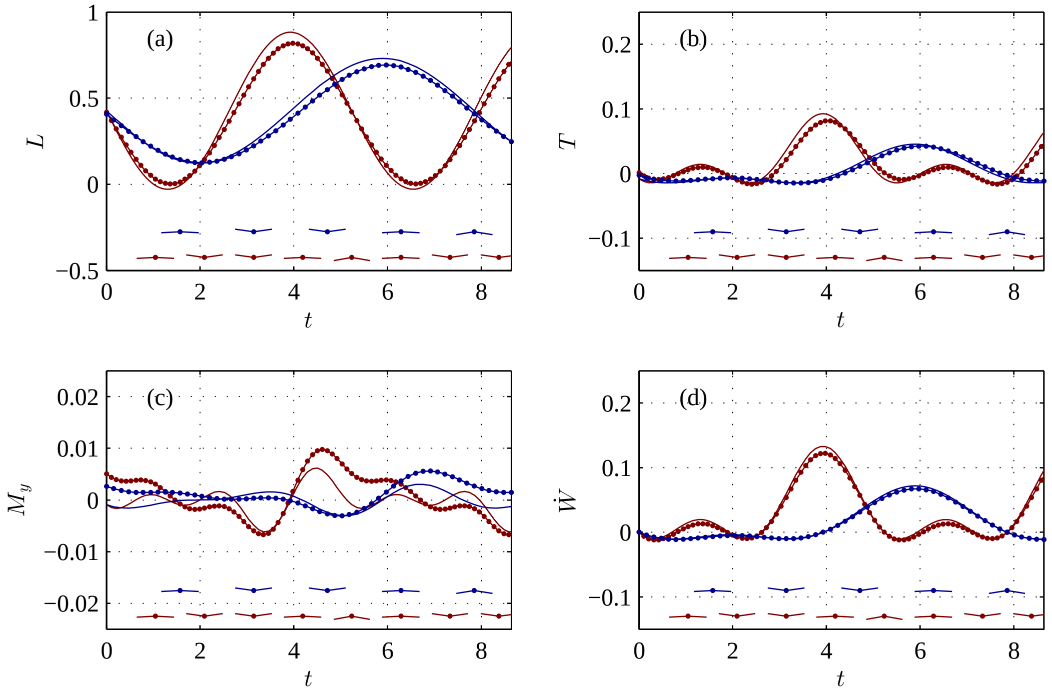

Figure S2.4: $A = 7$, $\phi_0 = 15$, $\varepsilon = 0.3$. $\omega = 0.8$ (blue) $\omega = 1.2$ (red).

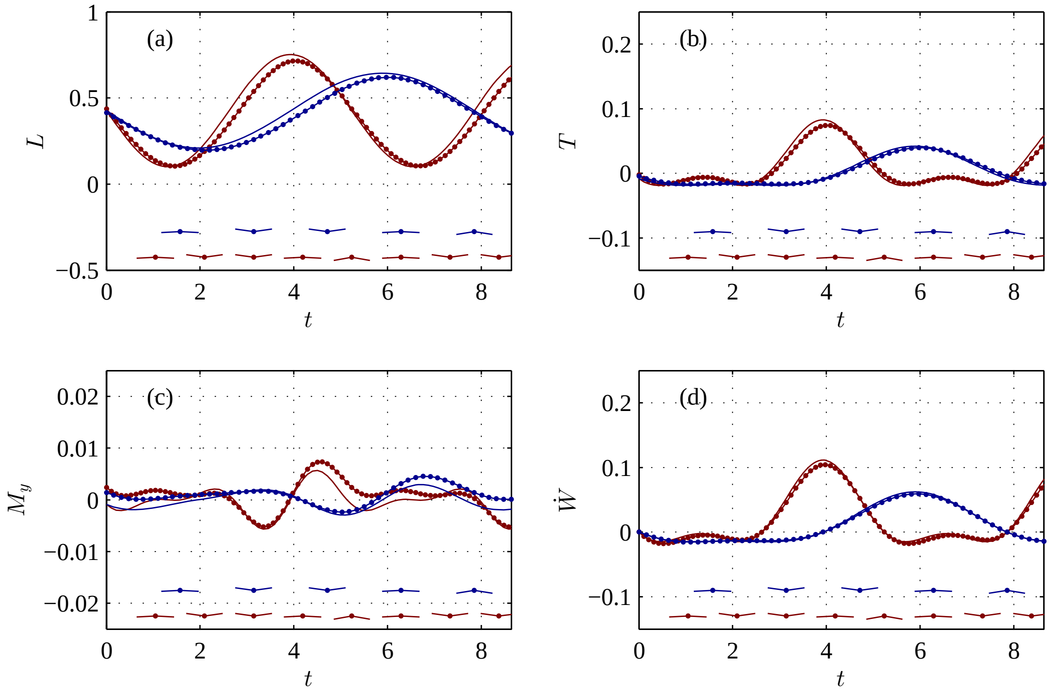

Figure S2.5: $A = 7$, $\phi_0 = 15$, $\varepsilon = 0.5$. $\omega = 0.8$ (blue) $\omega = 1.2$ (red).

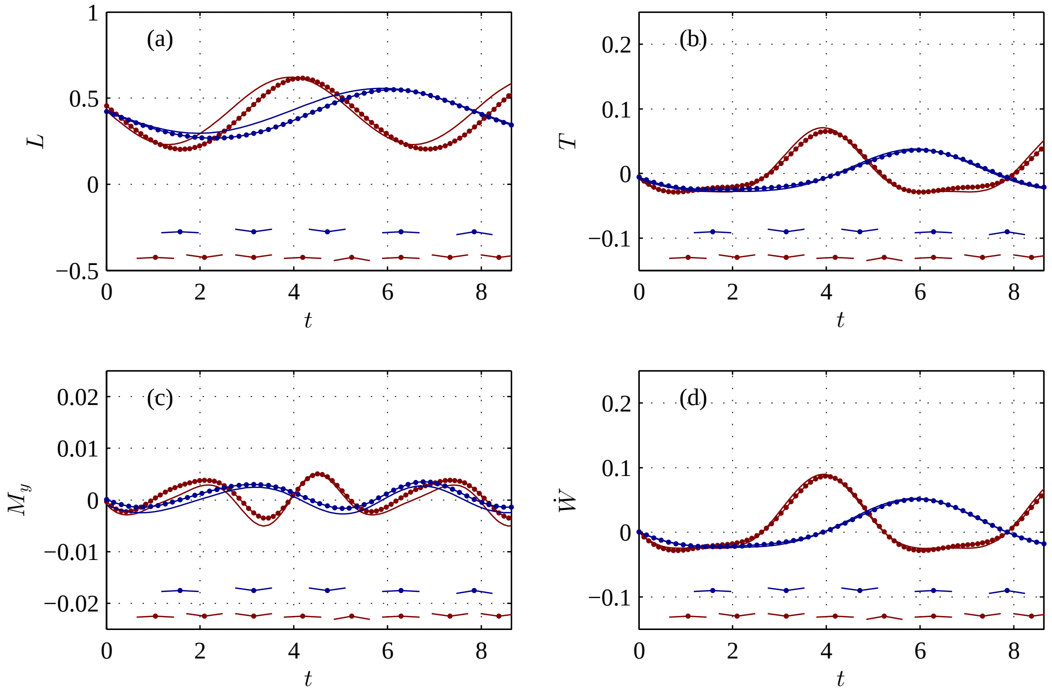

Figure S2.6: $A = 7$, $\phi_0 = 15$, $\varepsilon = 0.7$. $\omega = 0.8$ (blue) $\omega = 1.2$ (red).

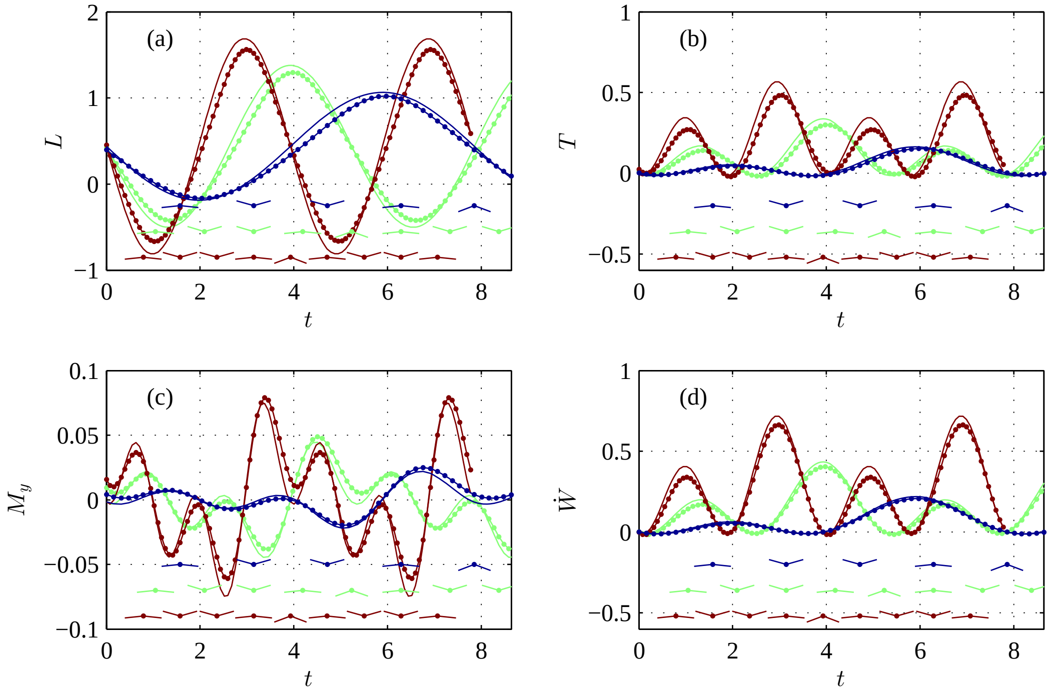

Figure S2.7: $A=8$, $\phi_0=30$, $\varepsilon=0.3$. $\omega=0.8$ (blue) $\omega=1.2$ (green) and $\omega=1.6$ (red).

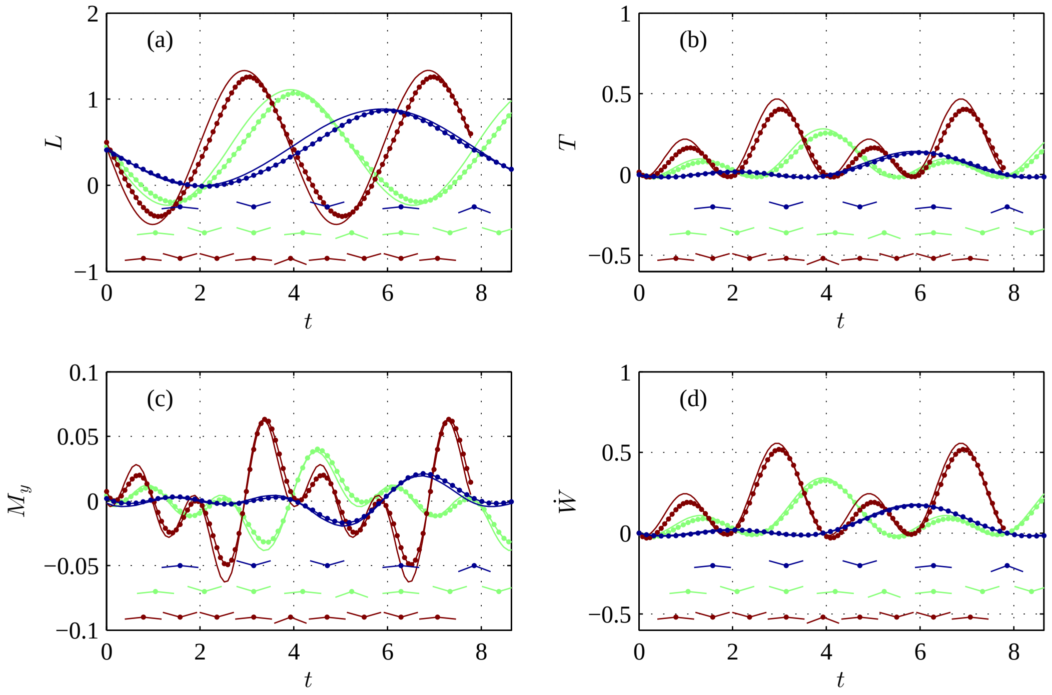

Figure S2.8: $A=8$, $\phi_0=30$, $\varepsilon=0.5$. $\omega=0.8$ (blue) $\omega=1.2$ (green) and $\omega=1.6$ (red).

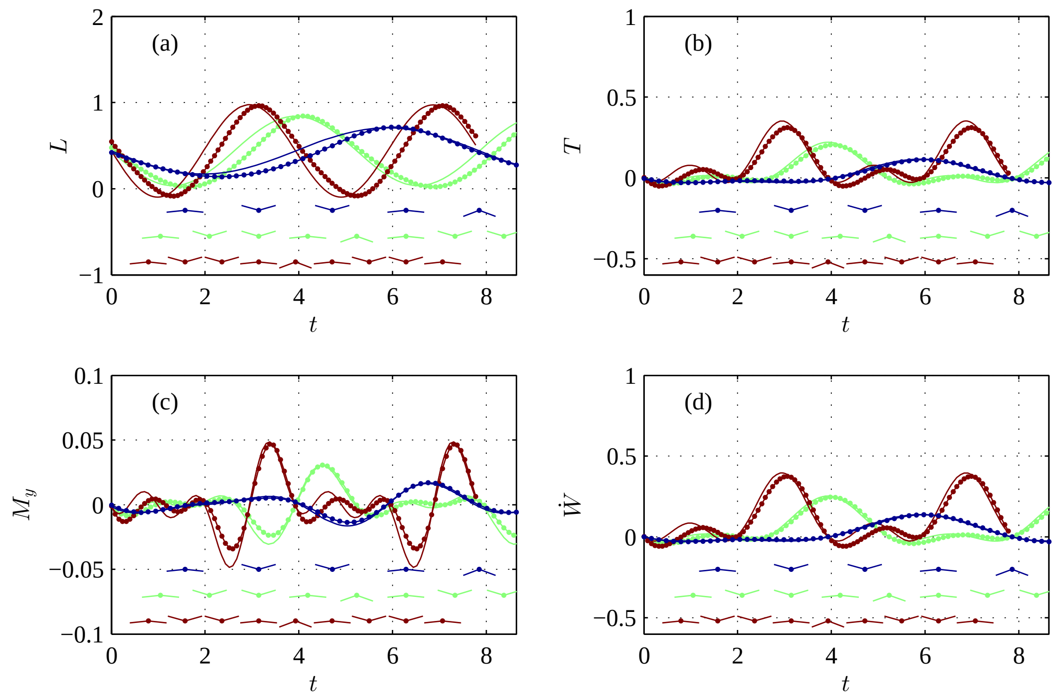

Figure S2.9: $A = 8$, $\phi_0 = 30$, $\varepsilon = 0.7$. $\omega = 0.8$ (blue) $\omega = 1.2$ (green) and $\omega = 1.6$ (red).

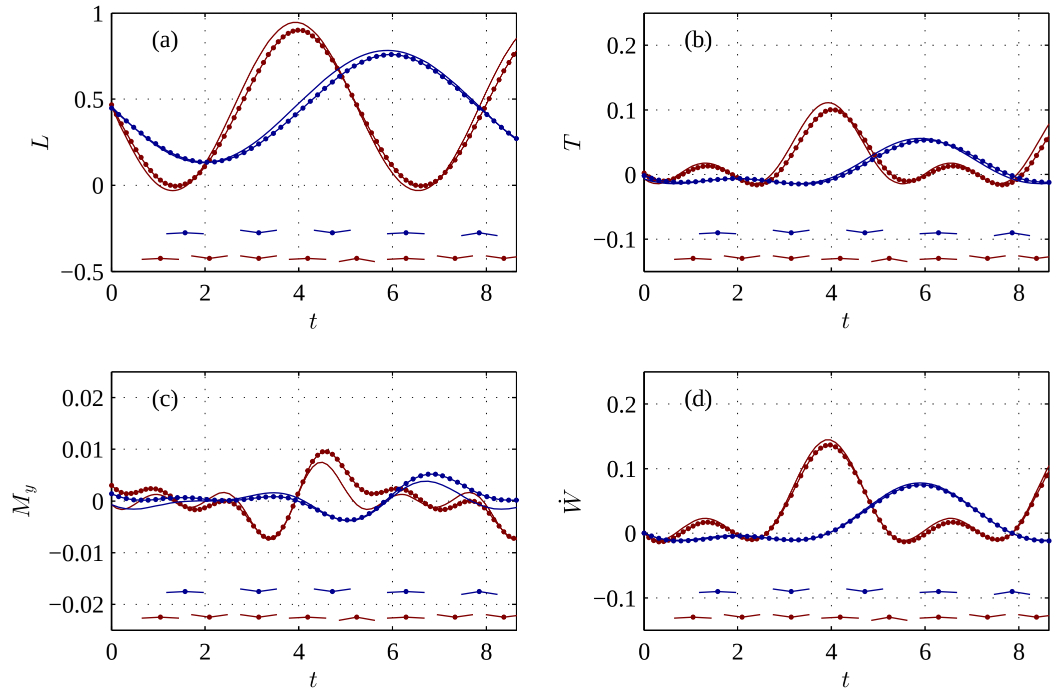

Figure S2.10: $A = 10$, $\phi_0 = 15$, $\varepsilon = 0.3$. $\omega = 0.8$ (blue) and $\omega = 1.2$ (red).

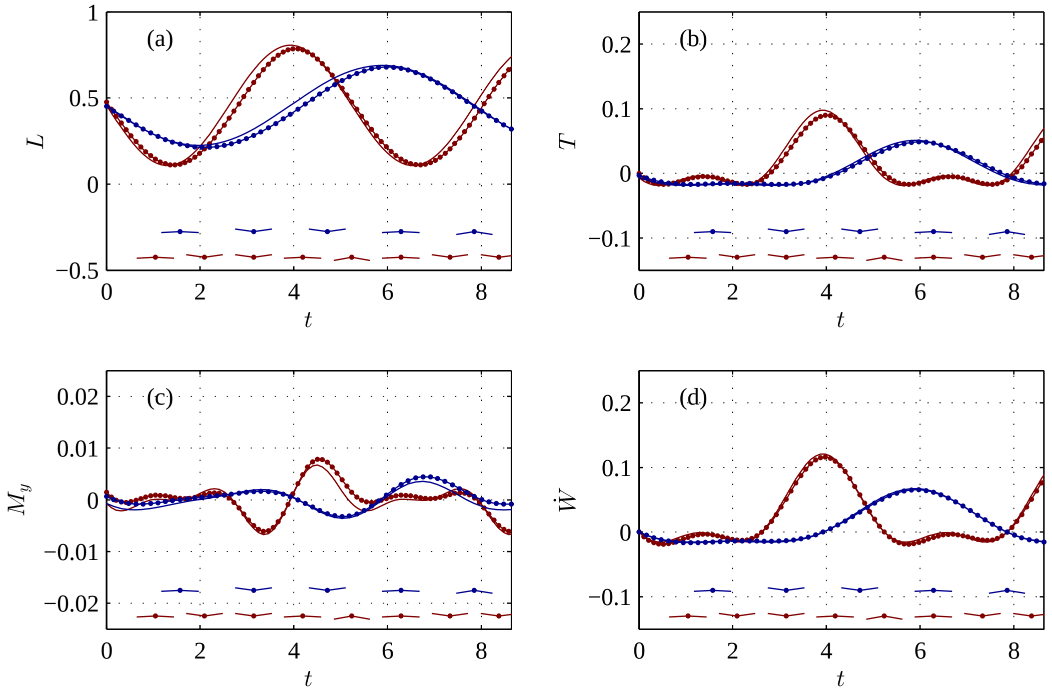

Figure S2.11: $A = 10$, $\phi_0 = 15$, $\varepsilon = 0.5$. $\omega = 0.8$ (blue) and $\omega = 1.2$ (red).

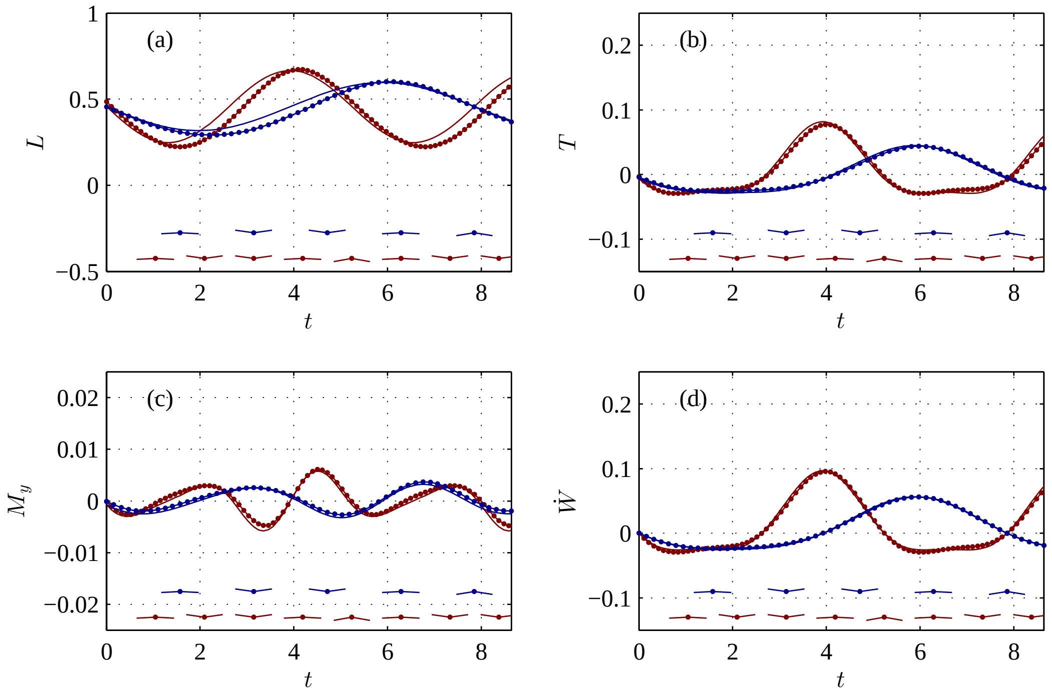

Figure S2.12: $A = 10$, $\phi_0 = 15$, $\varepsilon = 0.7$. $\omega = 0.8$ (blue) and $\omega = 1.2$ (red).

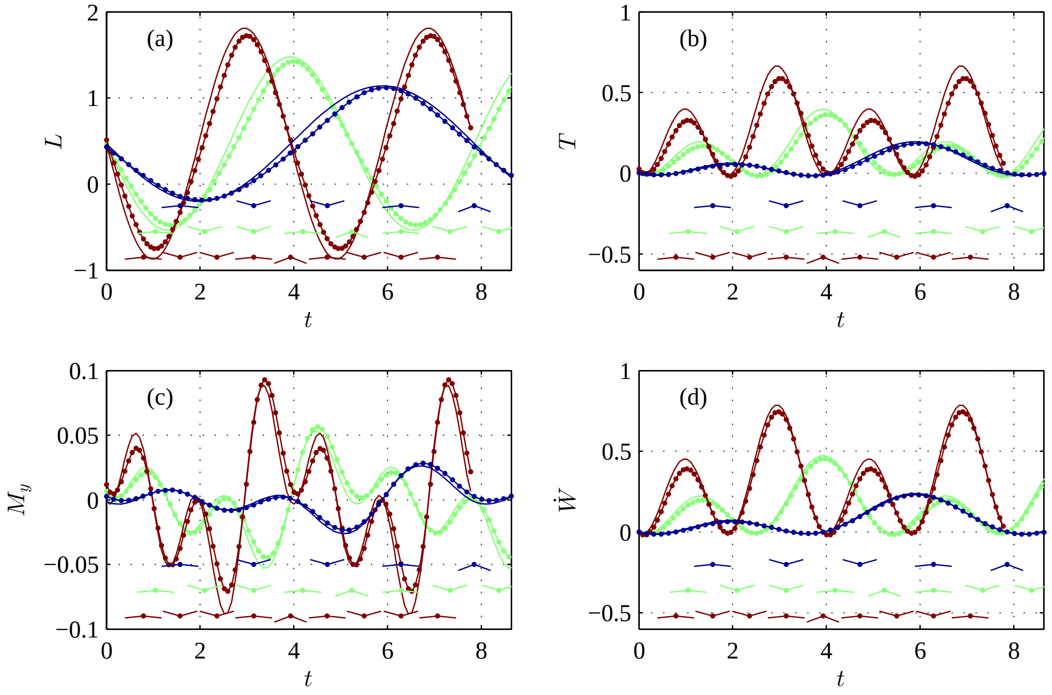

Figure S2.13: $A = 12$, $\phi_0 = 30$, $\varepsilon = 0.3$. $\omega = 0.8$ (blue) $\omega = 1.2$ (green) and $\omega = 1.6$ (red).

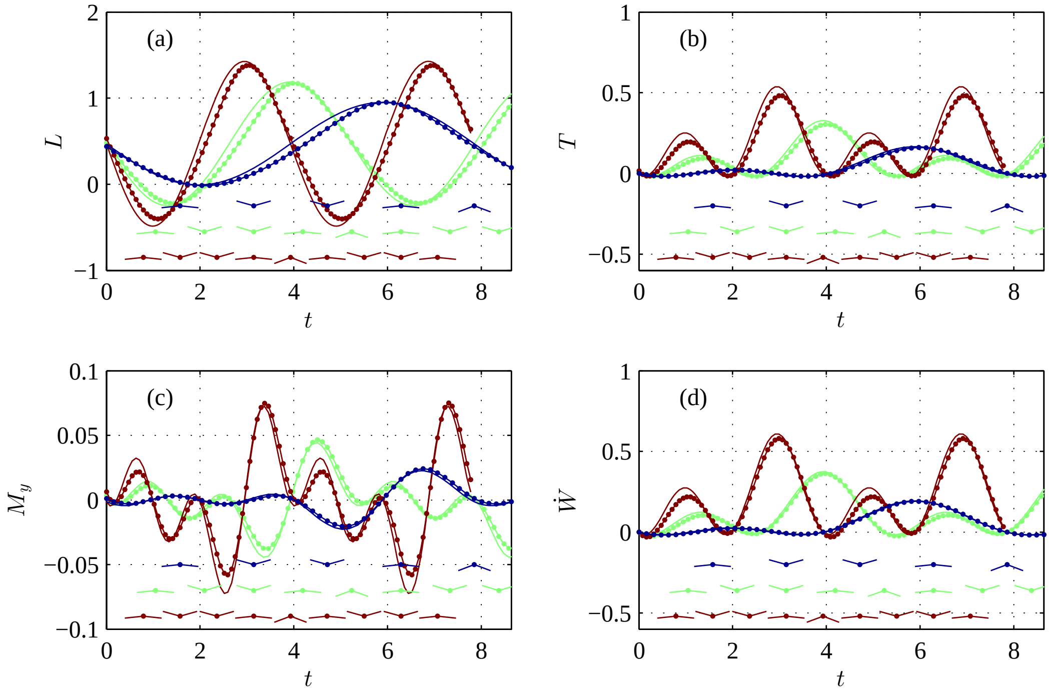

Figure S2.14: $A = 12$, $\phi_0 = 30$, $\varepsilon = 0.5$. $\omega = 0.8$ (blue) $\omega = 1.2$ (green) and $\omega = 1.6$ (red).

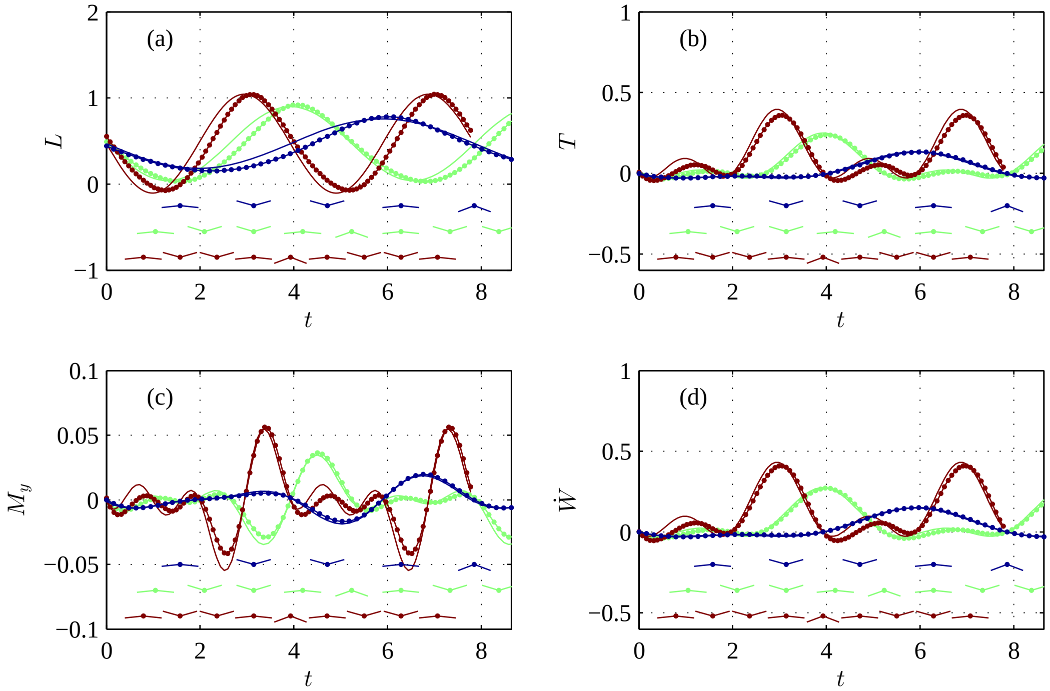

Figure S2.15: $A = 12$, $\phi_0 = 30$, $\varepsilon = 0.7$. $\omega = 0.8$ (blue) $\omega = 1.2$ (green) and $\omega = 1.6$ (red).

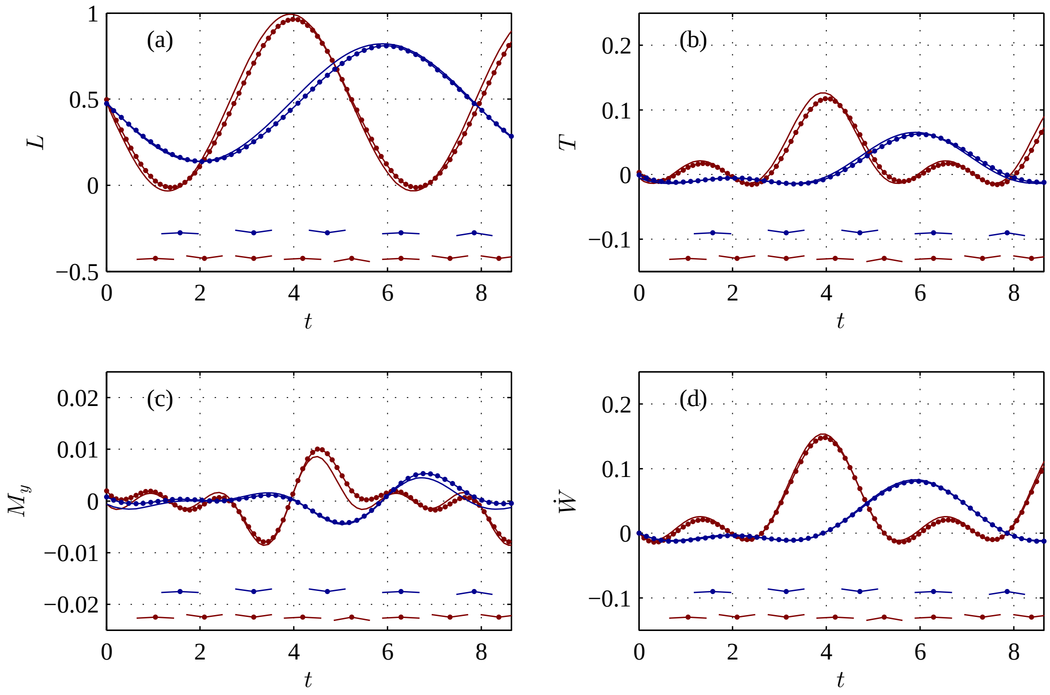

Figure S2.16: $A = 14$, $\phi_0 = 15$, $\varepsilon = 0.3$. $\omega = 0.8$ (blue) and $\omega = 1.2$ (red).

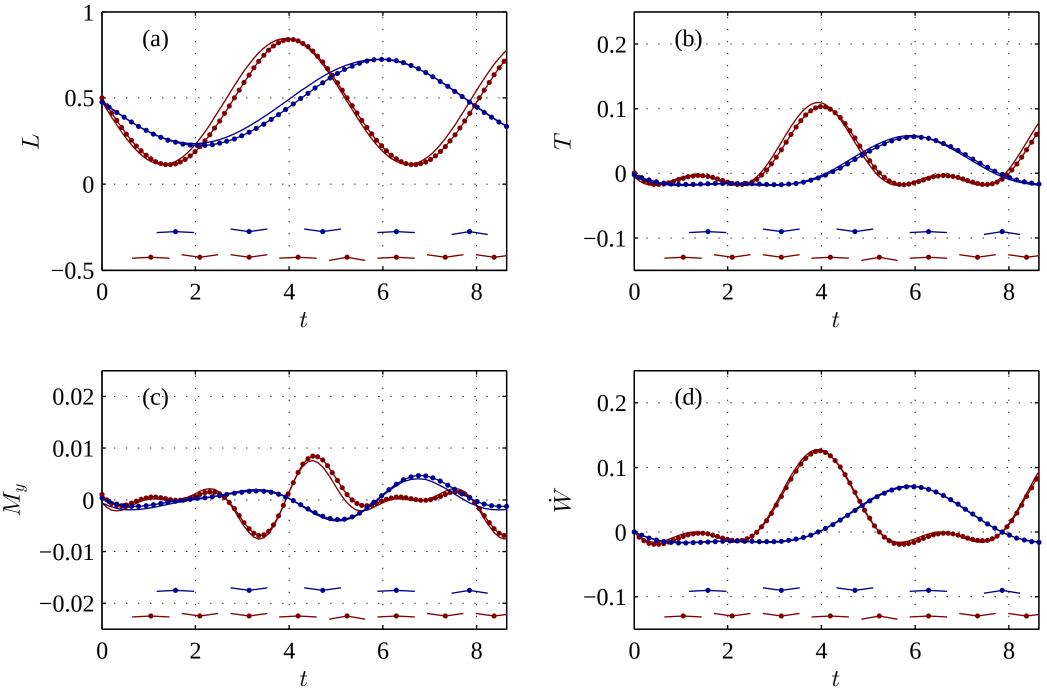

Figure S2.17: $A=14$, $\phi_0=15$, $\varepsilon=0.5$. $\omega=0.8$ (blue) and $\omega=1.2$ (red).

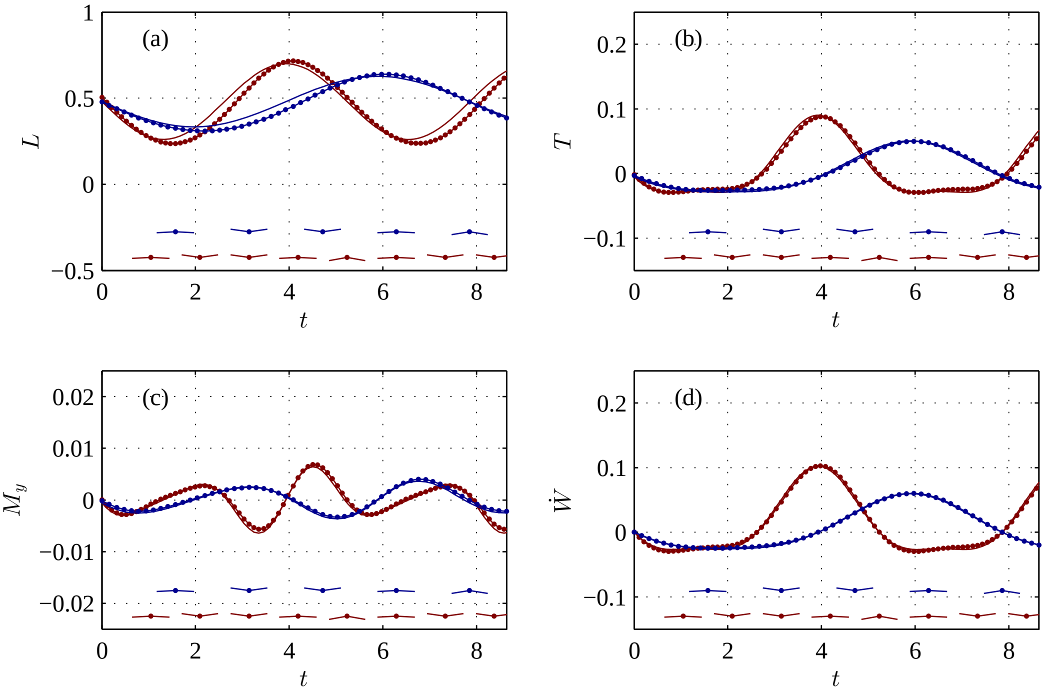

Figure S2.18: $A=14$, $\phi_0=15$, $\varepsilon=0.7$. $\omega=0.8$ (blue) and $\omega=1.2$ (red).

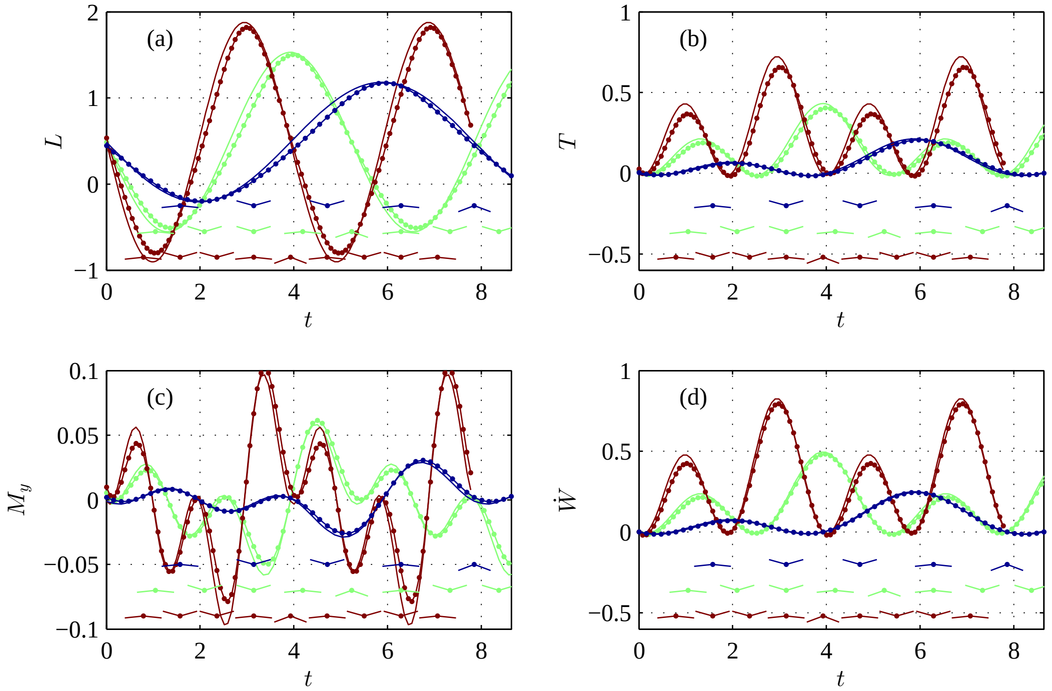

Figure S2.19: $A=16$, $\phi_0=30$, $\varepsilon=0.3$. $\omega=0.8$ (blue) $\omega=1.2$ (green) and $\omega=1.6$ (red).

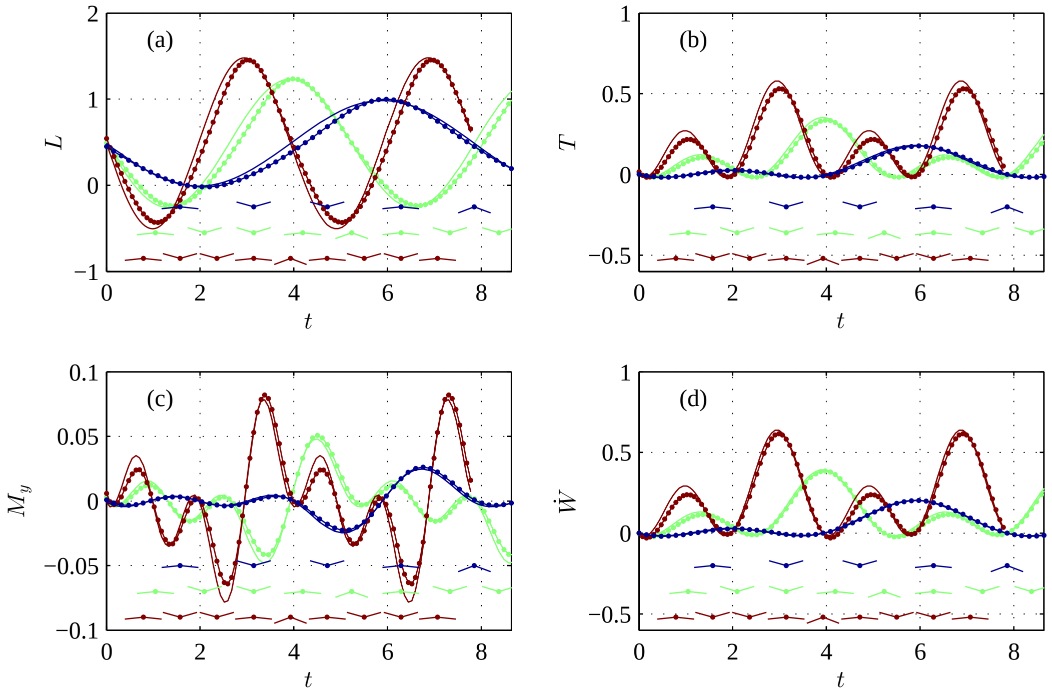

Figure S2.20: $A=16$, $\phi_0=30$, $\varepsilon=0.5$. $\omega=0.8$ (blue) $\omega=1.2$ (green) and $\omega=1.6$ (red).

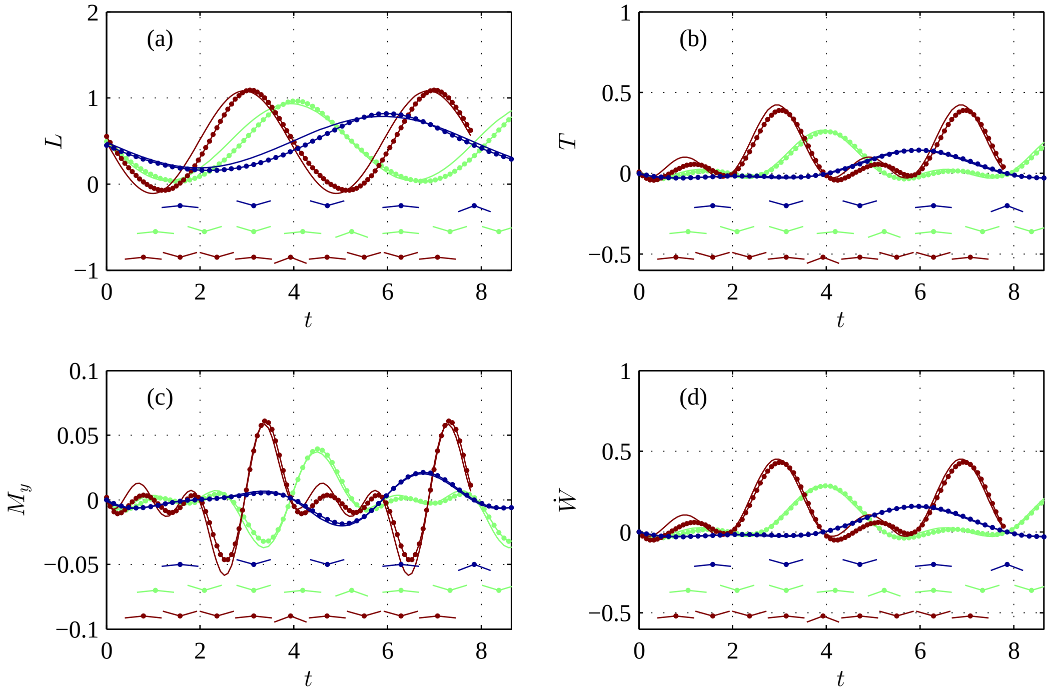

Figure S2.21: $A = 16$, $\phi_0 = 30$, $\varepsilon = 0.7$. $\omega = 0.8$ (blue) $\omega = 1.2$ (green) and $\omega = 1.6$ (red).
